# Diagnostic tools in Rhinology EAACI position paper

**DOI:** 10.1186/2045-7022-1-2

**Published:** 2011-06-10

**Authors:** Glenis Scadding, Peter Hellings, Isam Alobid, Claus Bachert, Wytske Fokkens, Roy Gerth van Wijk, Philippe Gevaert, Josep Guilemany, Livije Kalogjera, Valerie Lund, Joaquim Mullol, Giovanni Passalacqua, Elina Toskala, Cornelius van Drunen

**Affiliations:** 1 Royal National Throat, Nose and Ear Institute, London, UK; 2 University Hospitals Leuven, Belgium; 3 University Hospital Barcelona, Spain; 4 University Hospital Ghent, Belgium; 5 University Hospital Amsterdam, The Netherlands; 6 University Hospital Rotterdam, The Netherlands; 7 University Hospital Zagreb, Croatia; 8 University Hospital of Genoa, Italy; 9 Center for Applied Genomics, Philadelphia, USA

## Abstract

This EAACI Task Force document aims at providing the readers with a comprehensive and complete overview of the currently available tools for diagnosis of nasal and sino-nasal disease. We have tried to logically order the different important issues related to history taking, clinical examination and additional investigative tools for evaluation of the severity of sinonasal disease into a consensus document. A panel of European experts in the field of Rhinology has contributed to this consensus document on Diagnostic Tools in Rhinology.

## Introduction

There are several reasons for accurate investigation of upper airways disorders like allergic rhinitis [1] and rhinosinusitis [2]. The first reason relates to the fact that such problems impact very significantly upon patients' quality of life and that well directed treatment can ameliorate the impairment of quality of life. The second is that some of these disorders are severe with significant morbidity and even mortality, and that presentation often occurs in the upper airway. Early diagnosis and effective management can prevent serious consequences, like in Wegeners' granulomatosis. The third reason relates to the fact that upper respiratory tract problems exacerbate lower respiratory symptoms and may extend to involve the lower respiratory tract. The nose is an air conditioner; filtering, warming and humidifying over 10,000 liters of air daily before it progresses to the lungs.

The nasal passages and associated structures bear the brunt of environmental contact being the first site of allergen, microbial and particle deposition. As a consequence the upper airway is the location of a highly developed innate and adaptive immune system. Effective mucociliary clearance is vital for respiratory health as evidenced by the effects of defects such as primary ciliary dyskinesia (PCD) and cystic fibrosis (CF). Lower airways disease is often preceded by nasal and sinus disease leading to a window of opportunity for early diagnosis and possibly prevention of severe complications. For example measurement of nasal nitric oxide is simple and quick and very low levels can alert the physician to the possibility of PCD before major lung damage is sustained, thus allowing the benefit of early physiotherapy.

Inflammatory airways diseases usually start in the nose. This observation does not only hold true for allergic and non-allergic rhinitis in older children and adults which can progress to asthma, but also for respiratory occupational disease, and for rhinosinusitis which can be the presentation of Wegener's granulomatosis or Churg Strauss syndrome and is also associated with bronchiectasis.

The ability to recognize and accurately diagnose nasal disease should be a part of the armamentarium of all allergists, chest physicians and paediatricians as well as ENT surgeons. In addition, the nose provides an ideal area for investigation of disease mechanisms. It has given us insights into the pathogenesis of allergic disease and of changes during pharmaco- and immunotherapy. Now investigations into other forms of inflammatory and non-inflammatory nose and sinus disease are ongoing with possibly new forms of therapy as a result. So researchers might also find this Position Paper of use. This document aims to provide a basic introduction into methods used in Rhinology - their applicability, specificity and sensitivity. It will hopefully become outdated soon by new advances in the field.

## History of the Patient

### Rationale

The patients' history is vital in understanding and diagnosing the problem. In rhinitis and rhinosinusitis an accurate history is usually more important than any other investigation.

The aim of any history taken is to evaluate the presence, severity and duration of symptoms, aiming at an accurate diagnosis and enabling adequate treatment.

### Definition

The medical history represents the patients' or a responsible carers' account of the problem, supplemented by direct questioning.

### Technique

A one-to- one interview is preferred, with or without the aid of a questionnaire with evaluation of the severity of the symptoms on a Visual Analogue Scale (VAS).

In spite of the patients' history being the mainstay of clinical diagnosis and therapeutic approach, the history and clinical examination in allergic rhinitis may deviate considerably from concordant SPT and sIgE results [3]. Patients' history is however the primary standard used in judging test sensitivity and specificity.

#### 1. Allergic Rhinitis

Rhinitis is defined as having two of the listed symptoms for >1 hour/day for >2 weeks: blockage, running (including postnasal drip), sneezing and itching. Nasal problems are often multi-factorial in nature, which needs to be taken into account when using the classification or considering treatment.

The diagnosis of allergic rhinitis (AR) is based upon the concordance between a typical history of allergic symptoms and diagnostic tests. Typical symptoms of AR include rhinorrhoea, sneezing, nasal obstruction and pruritus. Depending on the sensitization pattern, patients with AR may be predominantly runners and sneezers or suffer from chronic obstruction with discharge which is mainly post nasal. Ocular symptoms are common, in particular in patients allergic to outdoor allergens. Conjunctivitis is found in 70% of pollen allergic rhinitis patients and around half of those with perennial rhinitis. Always ask about the following eye symptoms: redness, discharge, itching, and vision impairment. Symptoms related to reduced smell and taste are more typical of rhinosinusitis.

**Figure 1:**
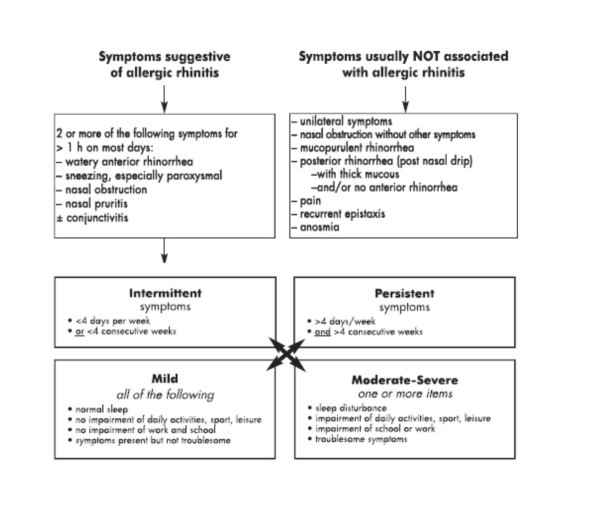
**Symptom-based diagnosis of allergic rhinitis**.

Figure 1 highlights the differences between symptoms suggestive of AR and those usually not associated with AR, and shows the criteria for severity of disease in relation to duration and associated extra-nasal symptoms. History should include specific symptom-related questions like:

• timing of symptoms (intermittent vs persistent disease, Figure 1)

• severity of symptoms (mild, moderate or severe, Figure 1)

• provoking factors e.g. animal contact

• alleviating factors e.g. holiday away from home

• occupational aggravation e.g. animal care facility

• seasonal aggravation e.g. grass pollen season

• effects of treatments tried in the past

• intolerance to medication e.g. aspirin

• associated oral allergy symptoms

Allergy is a more likely diagnosis if there is a past, present or family history of allergic diseases (AR, asthma, atopic dermatitis).

Rhinitis symptoms without obvious allergic triggers may still be allergic in origin so specific IgE testing is advisable for all sufferers. Those with a good history for an allergic cause who are negative on specific IgE tests in blood or on skin should have the benefit of a nasal challenge with the likely allergen since local nasal IgE production can occur.

AR and asthma usually co-exist, with symptoms of rhinitis found in 75-80% of patients with asthma and asthma in up to a third of rhinitis patients. Therefore patients should also be asked about lower respiratory tract symptoms (wheeze, cough, dyspnoea, sputum) and functional measurements (spirometry, peak flow) made.

There is an association between rhinitis and OME in childhood [4] so questions on hearing, listening, language, learning and behavior should be included in children with rhinitis, and ear examination and hearing tests including tympanometry and audiometry performed if a problem is suspected. Adults rarely develop OME unless they have a severe form of rhinosinusitis (Churg-Strauss syndrome, aspirin sensitivity, allergic fungal sinusitis).

Pharyngitis/laryngitis- can occur secondary to rhinitis or may be the predominant feature.

Food allergy is often associated with allergic airway disease and atopic dermatitis [5], and should therefore be asked for during history taking.

Sleep problems-are common in rhinitis and can be detrimental to quality of life and to work/school performance. Difficulty in going to sleep, snoring, mouth breathing at night, waking with a dry or swollen mouth and throat, daytime somnolence, headache, ability to attend and function at work or school should be queried.

#### 2. Non- Allergic rhinitis

**Figure 2:**
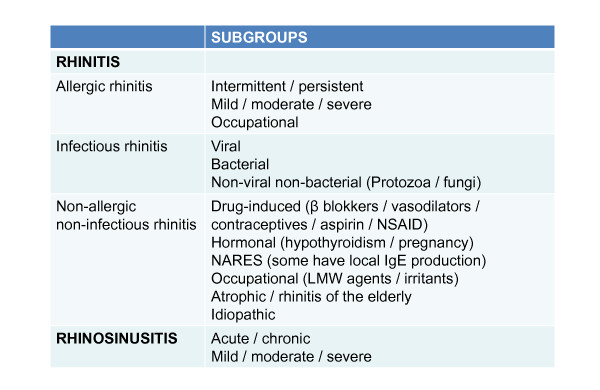
**Differential diagnosis of rhinitis/rhinosinusitis**.

In rhinitis patients who are not allergic, i.e. having negative skin prick test results or blood analysis for allergen-specific IgE, there is an extensive differential diagnosis (Figure 2).

In small children frequent viral upper respiratory tract infections occur- between 6 and 8 annually on average. Helpful questions include whether symptoms were present from birth (consider primary ciliary dyskinesia (PCD) or cystic fibrosis (CF)), and whether they ever remit (if not an immune defect is possible). Serious underlying disease is unlikely in children who are growing normally and who do not have problems beyond the upper respiratory tract.

In adults an extensive drug history may reveal overuse of topical alpha agonists or the possibility of aspirin or NSAID hypersensitivity that usually starts in adult life. The latter cannot be discounted if no such drug has been safely taken in recent months and a challenge may be necessary.

Hormonal rhinitis can occur, so questions about hormone therapy, possible thyroid auto-immunity, or pregnancy are needed.

Atrophic rhinitis can be a primary condition attributed to Klebsiella ozaenae or secondary to excessive surgery or radiation.

Neurogenic rhinitis is incompletely understood but is usually non-inflammatory, commoner in females and less likely to be associated with asthma. It may commence at a time of great stress. Old man's drip is thought to be hormonal since it responded to testosterone before therapy with ipratropium bromide was found to be effective.

#### 3 Rhinosinusitis

Rhinitis frequently co- exists with sinusitis, so the correct term in patients with symptomatic inflammation of the sinus cavities is rhinosinusitis.

Rhinosinusitis including nasal polyps (NP) ^2 ^is defined as inflammation of the nose and the paranasal sinuses characterized by two or more symptoms, one of which should be either nasal blockage/obstruction/congestion or nasal discharge (anterior/posterior nasal drip), +/- facial pain/pressure, +/- reduction or loss of smell; and either endoscopic signs of polyps and/or mucopurulent discharge primarily from middle meatus and/or; oedema/mucosal obstruction primarily in middle meatus, and/or Computerised Tomography (CT) changes showing mucosal changes within the ostiomeatal complex and/or sinuses.

The EP^3^OS document proposes to define the disease according to the duration of symptoms:

Common cold/acute viral rhinosinusitis is defined as an acute rhinosinusitis lasting <10 days.

Acute (non-viral or bacterial) rhinosinusitis is defined by an increase in symptoms after 5 days or PER symptoms after 10 days with <12 weeks duration.

Chronic rhinosinusitis/NP is defined symptoms for >12 weeks.

The disease can be divided into MILD, MODERATE or SEVERE based on the total severity VAS score (0-10 cm): MILD = VAS 0-3; MODERATE = VAS 3.1-7; SEVERE = VAS 7.1-10.

As is the case with AR, patients with rhinosinusitis should be asked for the following:

• onset of symptoms (since birth, adolescence or adulthood)

• timing of symptoms (acute vs chronic disease)

• severity of symptoms (mild, moderate or severe)

• provoking factors e.g. microbial or occupational factors

• alleviating factors e.g. holiday away from home/work

• seasonal aggravation in allergic patients

• effects of treatments tried in the past

• intolerance to medication e.g. aspirin

• associated bronchial symptoms

• familial history of sinus disease (NP disease)

### Recommendations

Adequate time and attention should be given to take a complete and accurate history both of rhinitis symptoms and those of possible co- morbidities. The history should suggest further diagnostic tests needed for a proper diagnosis. All rhinitis patients should have specific IgE tests unless the history itself is diagnostic e.g. recurrent symptoms confined to a known pollen season with remission by avoidance.

A subgroup of patients with rhinitis symptoms need to be referred to an ENT specialist for nasal endoscopy. ENT referral is needed for:

- unilateral nasal problems

- nasal perforations, ulceration or collapse

- sero-sanguineous discharge

- severe crusting within the nasal cavity

- recurrent infection

- periorbital cellulitis (refer urgently)

- severe sleep problems

## Quality of Life Instruments in Rhinology

### Rationale

The importance of quality of life issues in nasal disease has been well recognized. The effects of disease on daily functioning, work, leisure and school as perceived by the patient are considered as an important characteristic of rhinitis severity ^1^. Moreover, assessment of quality of life is one of the standard outcome measures in clinical trials acknowledging the fact that the classical outcome variables may only partially characterize the disease of the patient.

### Definition

Health related quality of life has been defined as "the functional effects of an illness and its consequent therapy upon a patient, as perceived by the patient [6]. Quality of life instruments aim to describe these effects. The patient's perspective is particularly important.

### Generic and disease-specific questionnaires

In general, two types of instruments are available, generic and disease-specific questionnaires. Generic questionnaires measure physical, psychological and social domains in all health conditions irrespective of the underlying disease. Those questionnaires allow the comparison between healthy and diseased subjects. Disease-specific instruments have been designed by asking patients what kind of problems they experience from their disease. Both the frequency and the importance of impairments are measured by means of the questionnaires. These instruments have the advantage that they describe the disease-associated problems of the patients.

There are important differences in the use of these instruments. Specific questionnaires have better discriminative and evaluative properties. On the other hand specific and generic instruments might cover different aspects of disease [7]. Moreover, an important characteristic of generic instruments is the ability to measure across diseases, thereby allowing for comparisons between different disorders. Finally, some generic questionnaires such as the Euroqol 5D [8] have been developed for cost-effectiveness studies.

### Usage of instruments

#### Clinical trials

Both generic and disease specific can be used in clinical rhinitis and rhinosinusitis trials. The responsiveness to change seems to be better with disease specific instruments. Generic instruments are less effective with mild disease [9]. From the many generic instruments the Short Form-36 health survey (SF-36) [10] and SF-12 [11] are commonly used in rhinitis and rhinosinusitis. In rhinosinusitis the McGill pain questionnaire [12] and the Glasgow benefit inventory (GBI) have been applied in several trials. The most frequently used rhinitis specific instruments [13] are the RQLQ [14] and its variations for adults (standardized RQLQ, mini-RQLQ [15]) and other age groups and Adolescent RQLQ [16]. A series of instruments has been developed to assess rhinosinusitis. From these questionnaires the Rhinosinusitis Disability Index (RSDI) [17] and in particular the SinoNasal Outcome Test 20 (SNOT-20) [18] are the most common outcome measures.

#### Cost-effectiveness studies

Cost-effectiveness studies use specific instruments designed for this kind of analysis. The Euroqol-5D [19] is a generic measure of health status that provides a simple descriptive profile and a single index value that can be used in the clinical and economic evaluation of health care. The (RSUI) was developed as a disease specific preference-based measure of rhinitis symptoms, also to be used in cost-effectiveness studies [20]. To date, there are no cost-effectiveness trials in patients with rhinitis or rhinosinusitis using these instruments. This can be attributed to a lack of cost-effectiveness studies in this area.

#### Clinical practice

Ideally, clinicians should be able to estimate the burden of disease in their patients. A quality of life questionnaire might be helpful. Many HQLQ instruments however are developed for use in clinical trials. In a recent systematic review 13 disease specific HQLQ tools for adults were evaluated [21]. One questionnaire, the Rhinasthma [22] evaluates patients with rhinitis and asthma. Several questionnaires can be used in practice [23-31] (see table 1).

**Table 1:** Instruments used in allergic rhinitis and in chronic rhinosinusitis

		Generic	Disease specific
Allergic rhinitis			

	Children		Pediatric RQLQ, adolescent RQLQ

	Adults	SF-36, SF-12, 15D [23], EuroQol 5D	RQLQ, standardized RQLQ, mini-RQLQ, Nocturnal Quality of Life Questionnaire (NQLQ) [24], Rhinitis Outcome Questionnaire [25] #, Rhinitis Symptom Utility Index (RSUI)

Chronic rhinosinusitis			

	Children	(CHQ) [26]	SN-5 quality of life survey [27]

	Adults	SF-36, SF-12, McGill pain questionnaire (MPQ), EuroQol 5D, Glasgow benefit inventory (GBI)	Rhinosinusitis Outcome Measurement (RSOM-31) [18] #, Rhinosinusitis Disability index (RSDI) #, sinonasal outcome test 16 (SNOT-16) [28] #, SNOT-20#, Chronic sinusitis survey (CSS) [29], RhinoQol [30], Sinusitis outcomes questionnaire (SOQ) [31]

# for use in clinical practice

##### Rhinitis control

Quality of life will improve if the disease is well controlled. HRQL questionnaires, however, do not estimate to what extent a disease is under control. In recent years new tools for asthma control have been developed and validated. Rhinitis tools are being developed, but not published yet.

### Evidence based instruments

The development of HQLQ instruments comprises a set of procedures for validation, determination of reliability and responsiveness. In general, these tools are better studied than classical outcome measures. However, in a recent systematic review of the quality of disease specific HQLQ four instruments only were identified as adequate in terms of discriminant validity (important for cross-sectional analysis) and responsiveness (important for longitudinal studies). For rhinitis the RQLQ and standardized RQLQ and for rhinosinusitis the RSOM-31 and the RhinoQOL appeared to be effective [18].

### Recommendations

The choice for an instrument depends on its purpose and the target population. For purposes of research other questionnaires are needed than for the evaluation of patients in clinical practice. The above-mentioned review gives some guidance to clinicians interested in the evaluation of quality of life in patients affected with AR and RS [21]. It has been suggested that the use of both generic and specific instruments may be useful [7], although this may not be always the case. As the outcome of quality of life assessment is only partly associated with clinical outcome measures, it is recommended to evaluate patients with both HRQL and medical measures.

## Nasal Examination

### I. GENERAL INSPECTION

#### Rationale

**Figure 3:**
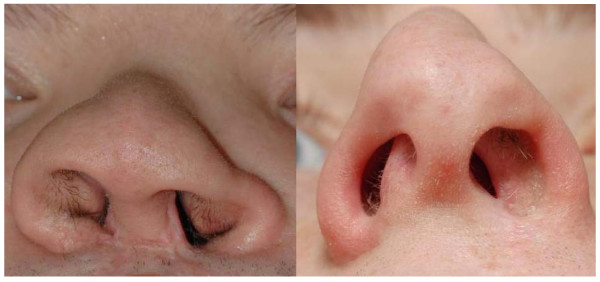
**Inspection of the nose showing distortion of the anatomy at the level of the nasal entry (in cleft lip patients)**.

**Figure 4:**
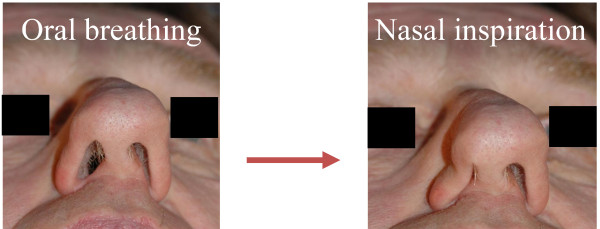
**Inspection of the nose showing alar collapse during inspiration**.

In the evaluation of a patient with (sino-)nasal symptoms, it is indispensable to start with a good inspection of the nose and face, both during inspiration as well as during expiration. Major anomalies can be visualized directly, like nasal vestibulum stenosis in cleft lip patients (Figure 3), collapse of the nostrils during inspiration (Figure 4) or severe septal deviations. Therefore, the aim of inspection of the nose is to delineate any anatomical problems that can interfere with the nasal function both in rest as well as during inspiration.

#### Definition

Inspection is the visual investigation of the external structures of the nose and beyond, in order to get a first and superficial impression of the nose and nasal function.

#### Technique

During history taking and clinical examination, it is important to look at the nasal anatomy, both at rest as well as during inspiration. With the aid of a light source, one can even better evaluate the bony, cartilaginous and skin parts that constitute the nasal dorsum.

The following nasal aspects are evaluated:

- The shape: congenital or acquired abnormalities of the ossa nasalia, middle vault and external valve area. A widened dorsum of the nose can indicate the presence of nasal polyps, caused by the dispersing of the ossa nasalia by nasal polyps (Woakes Syndrome, typical for CF). A horizontal nasal crease across the dorsum of the nose supports a diagnosis of AR. The presence of vestibular stenosis (Figure 3), alar collapse at the time of inspiration (Figure 4) and/or narrow middle vault are associated with nasal obstruction and can be observed during external inspection of the nose during inspiration.

- The position: examination of the bony nose bridge, mostly deviated by trauma, can be hampered by the post-traumatic swelling. Examination of the cartilaginous tip of the nose, mostly deviated during growth.

- The covering skin of the nose: search for color changes, edema, skin lesions, fistulas or scarification.

- The surrounding structures: forehead, eyes, cheeks and upper lip.

### II. Palpation

#### Rationale

This simple and inexpensive act is a proper manner to trace pathology of the skin, the tissues, the bony and cartilaginous parts of the nose.

#### Objectives

To evaluate the nose with the fingertips, in order to search for shape or tissue anomalies, painful or sensitive areas, and/or lack of tip support mechanisms.

#### Definition

Examination of the skin, underlying tissues, the bony and cartilaginous parts for irregularities, abnormal mobility, pressure pain and tip support.

#### Technique/instrumentation

A proper light source is necessary for an accurate inspection. With palpation one can detect a nasal valve dysfunction, particularly with the Cottle test. The cheek of the evaluated side is gently pulled laterally with one or two fingers, which opens the valve. The examiner then asks the patient to breathe and then evaluates if breathing is subjectively better after pulling the cheek. A positive test result is when the patient feels less resistance with the valve opened. This test is easy and quick to perform, but has a high false positive result rate.

**Figure 5:**
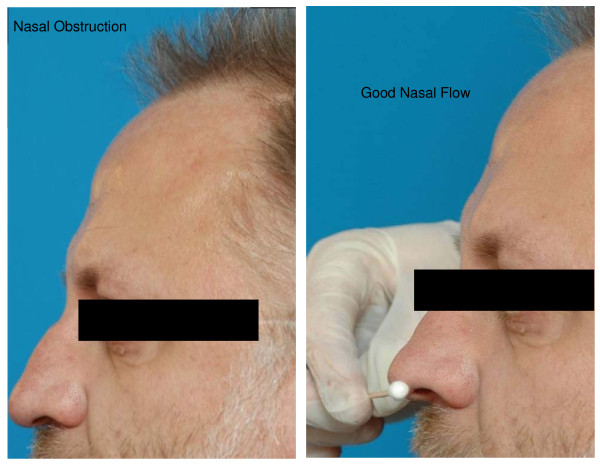
**Tip elevation test for evaluation of improved breathing by restoration of normal tip support**.

In case of lack of tip support, the tip elevation test (Figure 5) may provide the examiner with valuable information on the cause of nasal obstruction. The patient is asked for improvement of nasal breathing by holding the nasal tip in a position with a straight naso-labial angle as depicted in Figure 5.

## Anterior Rhinoscopy

### Rationale

Anterior rhinoscopy makes a quick but limited internal inspection possible of the anterior parts of the cavum nasi.

### Objectives

An inspection of the clinical status of the anterior parts of the nose. Trace nasal discharge or mucosal aberrations like swelling, crusting, mucosal perforations, large polyps.

### Definition

**Figure 6:**
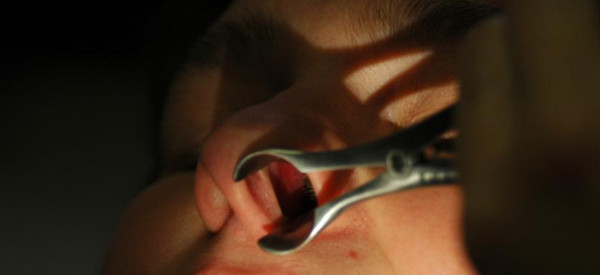
**Anterior rhinoscopy allowing the evaluation of mucosal and/or anatomic pathology at the anterior part of the nasal cavity**.

Internal inspection of the vestibulum and cavum nasi with the aid of an examination lamp fixed to a headband and a nose speculum (Figure 6).

### Technique/instrumentation

With a forehead light and nose or ear speculum. Without speculum, the tip of the nose can simply be pushed upwards and so, one can get a first impression of the position of the septum and of the head of the first concha (practical in case of examination of an infant). A right-handed observer takes the speculum in the right hand, while the left hand is used to position the head of the patient. The speculum, inserted under an angle of 45°, spreads the alar cartilages and pushes aside the hairs in the nose. The nasal septum must not be touched, because it is very sensitive. When the head of the patient is bend forward, the anterior part of the inferior meatus and concha can be visualized, by bending the head of the patient backwards, the anterior part of the middle meatus and concha. At last the speculum has to be removed closed to avoid avulsion of the hairs in the nose, which is very painful. It must not be forgotten to get a look in the oral cavity and pharynx for signs of pharyngitis, post nasal drip can cause lymphoid hyperplasia, resembling cobble stones.

**Figure 7:**
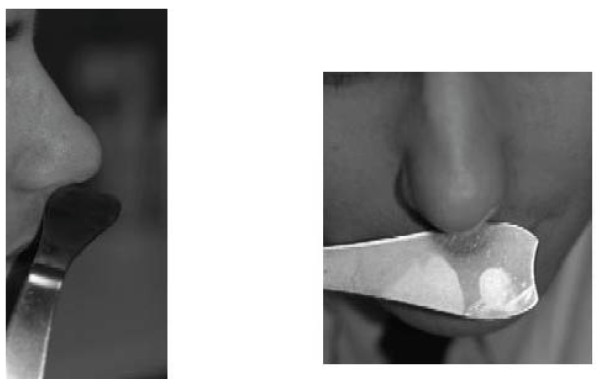
**Mirror test for evaluation of the condensate of expired air on a cold metal instrument or mirror**.

Nasal inspection can be supplemented by the so-called mirror test (Figure 7). By holding a cold mirror or small metal plate under the nostrils, the airflow during nasal expiration can be assessed. A lack of fogging indicates an inadequate nasal flow, or major asymmetrical fogging indicates unilateral obstruction.

### Sensitivity

Anterior rhinoscopy is limited in its evaluation of the entire nasal cavity. Therefore, complete and thorough examination using nasal endoscopy is advocated for patients with nasal symptoms. For example, small polyps may not be seen by anterior rhinoscopy.

### Outcomes

Possible clinical findings during anterior rhinoscopy are rhinorrhoe with transparent or discoloured secretions, asymmetries (mostly of the nasal septum), mucosal aberrations or edema, nasal polyps, neoplasms, corpora aliena, etc. One can assess the accessibility of the nose and the shape of the conchae.

## Posterior Rhinoscopy

### Rationale

This examination is performed to inspect the posterior parts of the cavum nasi, the choanae, the posterior parts of the lower and middle concha nasalis, the posterior septum, the nasopharynx with the adenoid and the ostia of the auditory tube.

### Definition

An inspection of the posterior parts of the cavum nasi and the nasopharynx with the aid of a small throat mirror.

### Technique/instrumentation

Posterior rhinoscopy is performed with a forehead light, a tongue spatula in the left hand and a small throat mirror in the right hand. Cooperation of the patient and skills of the observer are required. Firstly, the mirror has to be heated till body temperature, otherwise it will be dimmed. Then the tongue spatula is placed in the middle of the tongue base whereby the tongue will be pushed down gently. The mirror can be advanced towards the space beyond the uvula. The act has to be performed carefully because touching the pharyngeal mucosa will trigger a vomiting reflex. When the palatum molle is too stringent, ask the patient to breathe calmly by the nose whereby the palatum molle relaxes and so the view extends.

### Outcomes

Possible conditions are congenital choanal atresia, acute adenoiditis, irritation of the rhinopharynx, post-nasal discharge, antro-choanal polyps, and Thornwald cysts.

### Recommendation

At present, this examination is not routinely being performed, and is often replaced by nasal endoscopy.

## Nasal Endoscopy (rigid and flexible)

### Rationale

**Figure 8:**
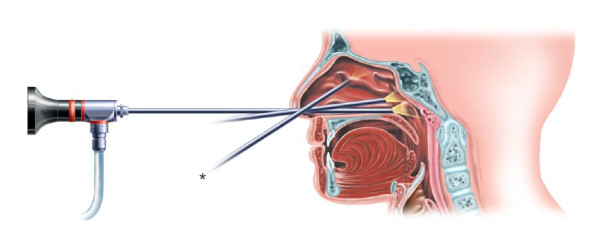
**Nasal endoscopy**.

In comparison with the anterior and posterior rhinoscopy, nasal endoscopy offers the advantage of global evaluation of the endonasal cavity (Figure 8).

### Objectives

Due to endoscopy, a good evaluation of the septum, the whole nasal cavity and the nasopharynx is possible, but also the area of the middle meatus which has clinical importance in rhinosinusitis.

### Definition

Nasal endoscopy allows inspection of the internal cavum nasi, with a bigger range of view and details in comparison with anterior and posterior rhinoscopy.

### Technique/instrumentation

Nasal endoscopy is performed by a flexible or rigid scope which is attached to a strong light source by glass fibre. For diagnostical examination, a scope with an optic angle from 25-30° is used with a calibre of 2,5-4 mm (Figure 8). Other optics are mostly used in surgery. Nasal endoscopy can eventually be preceded by local administration of anaesthetic drugs preferably in combination with a decongestivum. At first, the bottom of the nose unto the nasopharynx is to be inspected with an evaluation of the septum nasi, the lower turbinate, the choanae and the nasopharyx. Afterwards, the scope follows the edge of the middle concha towards the rostrum sphenoidale, with information about the middle and upper concha, the drainage from the sinuses, possible accessory ostia from the maxillary sinus and the aperture of the sphenoid sinus. At last, there must be attempted to get a view of the osteomeatal complex, the ethmoidal bulla and the access to the frontal sinus.

### Outcomes

Allergic and inflamed mucosa, secretions or swelling in the middle meatus, and possible presence of nasal polyps should be evaluated. Although the usefulness of nasal endoscopy in the evaluation of the nasal congestion is obvious, no clinical trials were found to support this statement. It was shown to be moderately sensitive and highly specific in predicting CT scanning results in patients with CRS [32]. Nasal polyps can be viewed with endoscopy, their presence and severity can be scored by validated systems with a good reproducibility. However, a correlation between size of polyps and the subjective symptom of congestion cannot be found. This discrepancy between objective findings and subjective complaints make endoscopy less suitable for assessment of severity. Still, when nasal polyps are present, nasal endoscopy scoring is very useful in treatment evaluation [33]. A possible semi-quantitative score for nasal polyps can be obtained at baseline and at regular intervals following therapeutic interventions (Table 2).

**Table 2:** Endoscopic appearance scores

Characteristic	Baseline and Follow-up
Polyp left (0,1,2,3)	

Polyp right (0,1,2,3)	

Oedema left (0,1,2)	

Oedema right (0,1,2)	

Discharge left (0,1,2)	

Discharge right (0,1,2)	

Postoperative scores to be used for outcome assessment only	

Scarring left (0,1,2)	

Scarring right (0,1,2)	

Crusting left (0,1,2)	

Crusting right (0,1,2)	

TOTAL POINTS	

Polyps: 0 Absence of polyps

1 Polyps in the middle meatus only

2 Polyps beyond the middle meatus but not blocking the nose completely

3 Polyps completely obstructing the nose

Oedema: 0 Absent; 1 Mild; 2 Severe

Discharge: 0 No discharge; 1 Clear, thin discharge; 2 Thick purulent discharge

Scarring: 0 Absent; 1 Mild; 2 severe

Crusting: 0 Absent; 1 Mild; 2 severe

## Diaphanoscopy of the *frontal and maxillary sinus*

### Definition

Transillumination of human tissue or a cavity, like a sinus, with a light source to evaluate the opacity of the hollow sinus.

### Technique/instrumentation

Transillumination of the maxillary sinus is performed with a light source in the mouth of the patient, watched in a darkened room. If the sinus is accessible (vacant), the light shines through the sinus and through the pupil. The frontal sinus can be investigated by diaphanoscopy if the light source is placed at the bottom of the frontal sinus. This examination is only useful in case of a unilateral acute maxillary or frontal sinusitis of an adult patient, who did not yet undergo sinus surgery.

Although the shortcomings of diaphanoscopy soon became apparent, the method was widely used for about half a century, but in the end could not compete with modern techniques of radiography and ultrasound [34].

## Recommendations

Inspection, palpation and anterior rhinoscopy are easy and rapid ways to examine a nasal problem without inconvenience to the patient. Therefore they should be the corner stone of every physical examination. Anterior rhinoscopy allows a limited internal inspection of the nasal cavity. In the majority of patients with persistent nasal symptoms, a complete and thorough examination of the nasal cavity is warranted using nasal endoscopy. For example, small polyps may not be seen by anterior rhinoscopy. Nasal endoscopy has been particularly useful in assessing the nasal airways in the region just below the olfactory cleft. Rigid endoscopy has proven to be more patient friendly and supplies a better image than flexible endoscopy. Patients awarded each type of scope a pain score on an analogue scale, according to the level of discomfort experienced, and the operator noted the number of structures seen. Significantly more structures were visualized with the rigid scope than the flexible scope. The pain scores were similarly in favor of the rigid scope, showing a trend to less discomfort [35].

## Allergy Tests Including Provocation

### Rationale and objectives of diagnostic tests in allergy

In the diagnostic process of allergic rhinitis we assume that allergen-specific IgE is the triggering factor of symptoms and of the underlying inflammatory process. Thus, the main goal of the diagnostic tests is to demonstrate both the presence and functional relevance of such IgE. In fact, the presence of specific IgE alone (sensitization) does not necessarily imply the existence of allergic symptoms, and there are a relevant number of individuals who are sensitized but who are not clinically allergic [36].

**Figure 9:**
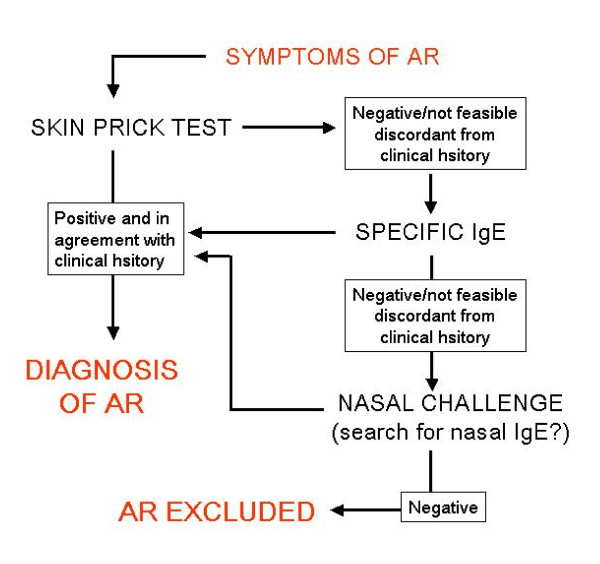
**Diagnostic algorithm for the diagnosis of AR**.

In patients with symptoms suggestive of AR, further diagnostic testing is required for optimal diagnosis and management (Figure 9). The presence of specific IgE can be demonstrated either *in vivo *(skin tests, SPT) or *in vitro *by detecting allergen-specific IgE in the blood (RAST, CAP-RAST and equivalent assays). Currently, SPT are unanimously considered the gold standard and the first-line approach for the detection of allergic sensitization, due to its' efficiency, safety and relatively low costs. The biological assays (CAP-RAST) have an equivalent efficiency, but due to costs are considered a second choice to be used only in special situations. The basophil degranulation tests require a special laboratory apparatus and, therefore, do not represent a routine option in the diagnostic workup. In addition, the functional role of specific IgE can be demonstrated in vitro by basophil activation tests or in vivo by the allergen-specific provocation.

**Figure 10:**
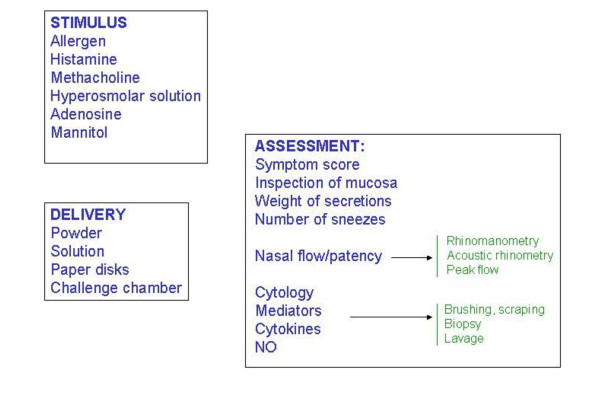
**Practical approaches for nasal provocation test**.

The nasal provocation tests aim at eliciting a nasal response, by delivering appropriate allergens to the nose. There is a wide variability of substances, measurements and evaluation techniques for nasal challenges (Figure 10). The purpose of the allergen specific provocation, is to reproduce at some extent the reaction occurring during the natural exposure to allergens. Thus, nasal allergen challenges allow demonstrating both the presence of allergen-specific IgE and the causal role of the allergen. The same happens with occupational substances or with aspirin, although in these cases the mechanism maybe non IgE-mediated. The non allergen- specific challenges evoke a non-specific inflammatory response, and demonstrate the existence of a nasal hyperreactivity.

### Skin prick test (SPT)

**Figure 11:**
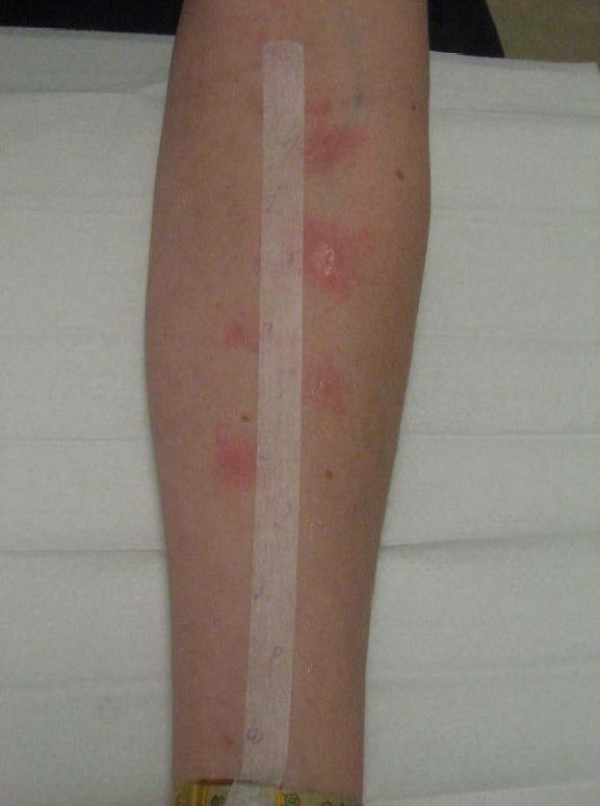
**Skin prick test with evaluation of wheal and flare reaction on the skin at the site of allergen deposition**.

The SPT technique is currently considered the gold standard method for the diagnosis of allergic rhinitis. With a trained investigator, they are highly reproducible [37,38]. Prick tests should be performed according to a rigorous methodology, with standardized diagnostic extracts, and always must include a negative (saline or diluent) and a positive control (histamine HCl 0.1%). Skin tests should be read at the peak of reaction (approximately at 15 minutes) by measuring the extension of wheals (Figure 11). The diagnostic and clinical significance of late reactions is not known. The scoring of the positivity is given according to EAACI recommendations, and the interpretation of a positive test must be integrated with the clinical history, since a positive SPT does not always imply a clinically relevant sensitization.

False positive reactions may occur, if a dermographism is present, but this can be ruled out with the use of the negative control.

False negative may occur due to:

a) weak potency of the extract;

b) inadequate technique (weak puncture);

c) interfering drugs. Systemic antihistamines are the most important drugs that reduce the skin reaction (Table 3). Thus, antihistamines must be discontinued at least 5 days before the SPT (for review see 11). On the other hand, antileukotrienes do not interfere with SPT and can be continued [39].

**Table 3:** Drugs affecting the results of skin tests

	Suppression	Duration of Suppression (days)
Cetirizine, desloratadine, ebastine, levocetirizine, mizolastine	++++	3-10

Chlorphenamine, promethazine	++	1-3

Ketotifen	++++	>5

Imipramine	++++	>10

Inhaled steroids	-	
Systemic steroids	+/-	

Cimetidine/ranitidine	-	

Antileukotrienes	-	

Intradermal tests are not of choice for the diagnosis of respiratory allergy, since they do not perform better than SPT and can induce false positive results. On the other hand, intradermal tests remain an essential part of the diagnostic workup for hymenoptera allergy [40]. The scratch test is no longer in use.

Atopy patch tests involve epicutaneous patch tests with allergens known to elicit IgE-mediated reactions. Commercial reagents are available for a few allergens, and have been standardized regarding the use of vehicle and dose-response relationships. A subset of patients with atopic dermatitis show only atopy patch test positivity while specific IgE to the same allergen remains negative [41], but the atopy patch test is usually not relevant for the diagnosis of respiratory allergies.

### Detection of allergen-specific IgE

The first method used for the measurement of serum allergen specific IgE has been the radioallergo sorbent test [42]. This has been now replaced by immune-enzymatic methods, including the widely used CAP assay. With these assays the level of specific IgE is expressed as kU/L, according to calibration curves, and the cut-off IgE level above which the test is positive is usually 0.35 KU/l. However, some sensitized subjects have an IgE level below this cut-off. The measurement of serum-specific IgE is usually less sensitive than skin prick tests [43] and the worst correlations between SPT and IgE assays are obtained with mold, food extracts and non standardized extracts. In general, the correlation between a strongly positive response to a skin test and the detection of serum-specific IgE and between a negative response to a prick test and the lack of detection of serum-specific IgE is very good. As in skin tests, the presence or absence of specific IgE in the serum does not imply a clinically relevant allergy. As mentioned above, for inhalant allergens, skin test responses represent the first-line diagnostic methods and when they correlate with the clinical history, in vitro tests are not necessary [1].

It has been suggested in the past that some patients may have a local IgE immune response without systemic IgE [44]. It has been recently shown that in a subset of patients the presence of specific IgE in the nasal mucosa can be demonstrated [45]. Nonetheless, the measurement of IgE in nasal secretions cannot be routinely proposed.

The presence of functionally relevant specific IgE can be demonstrated by putting the allergen into contact with basophils and subsequently detecting their activation by cytofluorometry. This basophil degranulation test has been proposed for specific conditions, such as drug allergies, but is not recommended at all for the diagnosis of respiratory allergies, and it is used only for research purposes.

Serum-total IgE is measured using either radioimmunoassay or enzyme assay. In normal subjects, levels of IgE increase from birth to adolescence and then decrease to reach a plateau after the age of 20-30 years. In adults, levels of over 100-150 KU/l are considered to be above normal. Nevertheless, an increase of total IgE correlates weakly with the presence of allergic diseases. Total IgE maybe increased in other conditions such as smoke and parasitic diseases. Thus, the measurement of total-serum IgE should no longer be used for screening or allergy diagnosis [1].

### Allergen specific nasal challenge (ASNC)

The ASNC procedure, also known as specific nasal provocation test (SNPT) or nasal allergen challenge (ANC) involves the delivery of a small quantity of the allergen into one (or both) nostril, in order to elicit the allergic reactions, if allergen-specific IgE is present in the nasal mucosa. By using progressively increasing amounts (or concentrations) of the allergen, a threshold dose can be also established. Recently, the availability of recombinant purified allergens has suggested the possibility to perform challenges with each specific allergenic protein, but the role of such approach in clinical practice is still not defined.

The procedure of allergen provocation was proposed more than one century ago and began to be systematically investigated starting from the 1950ties. Nowadays, there are some official documents available, where an attempt to standardize the procedure has been made [46]. The main advantages of ASNC are the simplicity of execution, the low cost and the safety. On the other hand, the procedure is still poorly standardized and the technical details (amount of allergen, interval between doses, dilutions, positivity criteria) are largely variable among centres. The main indications of ASNC are

a) to demonstrate the causal role of an allergen,

b) to identify the clinically relevant allergen(s) in polysensitized subjects,

c) to evaluate the effects of a treatment

d) to study the inflammatory phenomena (Table 4)

**Table 4:** Practical aspects of ASNC

PURPOSES	CONTRAINDICATIONS
Demonstration of the causal role of an allergen Identification of the most relevant allergen(s) Evaluation of the effect of a treatment Investigating the inflammatory phenomena (research) Occupational rhinitis	Acute bacterial or viral rhinosinusitis. Acute exacerbation of allergic disease. History of previous anaphylactic reaction Severe general diseases Pregnancy Polyps Recent ENT surgery (6-8 wks)
CAUSES OF FALSE POSITIVE	CAUSES OF FALSE NEGATIVE
Nasal cycle Recent exposure to irritants Rhinosinusitis Priming effect	Weak extract Drugs nasal antihistamine (1 day withdrawal) oral antihistamine (3 day withdrawal) nasal steroid (7 day withdrawal)

GENERAL PROCEDURE

Let the patient be adapted to room temperature

Inspect nasal cavity

Spray or apply saline as negative control

Perform measurement(s)

Instruct to avoid nasal breathing

Apply the allergen into one nostril and re-evaluate after 10-15 minutes

Proceed on with increasing concentration in the other nostril

Use both active and plasebo tests for diagnosis for occupational rhinitis

e) to evaluate the role of occupational allergens.

When an allergen is introduced into the nose, the IgE-mediate reaction immediately takes place and the classic symptoms appear within seconds. Symptoms slowly subside within 4-6 hours, may re-appear after several hours in case of a late phase reaction. The biphasic reaction can be easily demonstrated by nasal scrapings to assess the presence of the inflammation.

The SNPT can be evaluated in many different ways. The most common and practical is the measurement of the four classic nasal symptoms by an ordinal scale (0 = absent to 3 = severe), being the test positive if a cumulative score of 5 or more is obtained. Alternatively, a visual analog scale can be used. Another semi-quantitative evaluation is the weight of nasal secretions, but this is less practical and is used only in research settings.

A quantitative assessment of the ASNC can be made by instrumentally measuring the nasal flow or resistance, by nasal peak flow meter, acoustic rhinometry or rhinomanometry. These investigations will be dealt with further in the document.

Other possible modalities to evaluate the effect of allergen challenge are the assessment of the inflammatory infiltrate by nasal scraping/brushing (with differential cell count), or the measurement of specific mediators in nasal lavage, including tryptase, alpha2 microglobulin, albumin, leukotriens, interleukins, eosinophil cationic protein and others (36-40).

### Aspirin nasal challenge

The nasal challenge with aspirin is not truly an allergen challenge, since an IgE mediated mechanism is not involved. Nevertheless it has the value of a specific challenge and it is used to diagnose aspirin intolerance in the context of the aspirin hypersensitivity with respiratory manifestations. The nasal challenge with aspirin was introduced later than the oral and bronchial challenge [47], but has gained popularity since it rarely induces systemic reactions. Nasal aspirin challenge is used in patients with severe asthma in whom oral or bronchial aspirin challenges are contraindicated.

The aspirin challenge is sufficiently standardized and reproducible [48], although the possibility of false negative results exists and the negative predictive value is lower than the oral and bronchial challenges. For this reason, it is agreed that where an aspirin intolerance is suspected and the nasal challenge is negative, the oral challenge must be performed. The nasal aspirin challenge must be performed under medical supervision. Oral, nasal steroids and antileukotrienes should be discontinued at least 7 days before, whereas the withdrawal period is 3 days for antihistamines and 24 hours for decongestants and cromones. Lysine-aspirin solutions at 0.1, 1 and 2 M are used at 10-minute interval steps. The evaluation of the result can be either clinical (symptoms) or instrumental (acoustic rhinometry, anterior rhinomanometry), and pulmonary function must be monitored during the challenge.

### Non-specific nasal challenges

Nasal hyperreactivity is the capacity of the nasal mucosa to respond with clinical symptoms and inflammation to unspecific stimuli, which are not causing any mucosal reaction in normal subjects. Nonspecific nasal reactivity is common in patients with allergic rhinitis [49]. A wide variety of stimuli can be used to evoke nasal hyperreactivity. These stimuli may directly act on a single receptor such as histamine, adenosine monophosphate, and methacholine, or activate a more complex mechanism, such as mannitol, capsaicin, hyperosmolar solutions and cold air.

The results obtained with non-specific nasal challenges are often conflicting and difficult to interpret, due to the heterogeneity of methods, doses and outcomes. As an example, histamine and methacholine are both able to evoke a nasal reaction, that is more pronounced in subjects with rhinitis than in healthy controls [50], but only histamine is able to modify the nasal resistance. On the other hand, cold dry air was shown capable of differentiating between patients with perennial non-allergic rhinitis and healthy subjects, but histamine did not [51]. In addition, mannitol nasal challenge seems unable to activate mast cells, although a dose-response in eliciting symptoms has been reported for this test [52].

The adenosine monophosphate challenge is relative simple and reproducible and more sensitive than histamine challenge [53]. It has been proposed to predict the response to nasal steroids and as a surrogate marker to evaluate the anti-inflammatory effects of drugs.

Nasal capsaicin challenge displays a dose-dependent response [54], and is able to detect the nasal hyperreactivity in seasonal allergic rhinitis. Due to its selectivity for sensory nerves, it is mainly used for experimental purpose to study the cough reflexes.

The nasal provocation with cold dry air requires a special apparatus, and is currently used only for research purposes. Interestingly, the cold air provocation has been reported to be able to discriminate between rhinitis alone and rhinitis associated with asthma [55], and between non-allergic rhinitis and healthy subjects [51].

A threshold dose discriminating healthy and diseased subjects has not been univocally established, as happens for instance in asthma. Another problem with those tests is that only few of them has been sufficiently standardized. Finally, the role of nonspecific hyper-reactivity in distinguishing different forms of rhinitis has not been established yet. Thus, for the clinical purpose and diagnosis of allergic rhinitis, the use of non-specific tests is not essential.

## Assessing the sense of Smell

### Rationale

Several patients with rhinitis and/or rhinosinusitis complain of smell dysfunction, and treatment for these conditions aims at restoring olfaction.

### Objectives

To objectively evaluate the capacity of an individual to smell environmental odours.

### Techniques

Several techniques are currently available for the objective evaluation of an individuals' smell capacity. The different tests that have been reported in the literature are listed below and extensively described in the Appendix, with emphasis on their clinical use, validation and strengths and weaknesses.

### List of different diagnostic smell tests (Appendix 1)

University of Pennsylvania Smell Identification Test (UPSIT)

Connecticut Chemosensory Clinical Research Center Test (CCCRC)

Smell diskettes test

Odourant confusion matrix

Dutch odour identification test (GITU)

YN-odour Identification Test (YN-OIT)

T&T Olfactometer

San Diego Odor Identification Test (SDOIT)

Cross-Cultural Smell Identification Test (CC-SIT)

Combined olfactory test (COT)

Sniffin'-Sticks

Candy smell test (CST)

Alcohol Sniff Test (AST)

Culturally Adjusted University of Pennsylvania Smell Identification Test (CA-UPSIT)

Kremer smell test

Scandinavian Odour-Identification Test (SOIT)

Pocket Smell Test [56]

Eloit and Trotier Olfactory Test

Ramdon Test

Four-minute odour identification test

Barcelona Smell Test (BAST-24)

Nez du Vin smell test

### Recommendations

Smell testing should be an integral part of the diagnostic approach in patients with smell dysfunction, i.e. hyposmia, parosmia or anosmia as presenting symptoms of sino-nasal disease, post-traumatic or post-viral smell disorder. Smell dysfunction is a cardinal symptom of rhinosinusitis with/without nasal polyps, but novel data suggest that also allergic rhinitis is often associated with a subjective reduction in smell capacity.

## Assessing the Sense of Taste

### Objectives

To evaluate the capacity of taste of five basic taste sensations, i.e. salt, bitter, sour, umami and sweet, in patients complaining of dysfunction of smell and taste. Smell disorders may be associated with disturbed taste capacity, hence necessitating the evaluation of taste capacity in addition to smell capacity in these patients.

### Techniques

Gustometry with application of taste substances and electrogustometry are the methods of taste examination. There are various ways of applying taste substances during gustometry examination. The stimuli used in gustometry are: citric acid or hydrochloric acid (sour taste), caffeine or quinine hydrochloride (bitter taste), sodium chloride (salty taste), saccharose (sweet taste), monosodium glutamate (umami taste). Electrogustometry, widely used by clinicians to examine taste sensitivity, allows estimating the functioning of taste by means of electric excitability thresholds determined through the response to the irritation of taste buds area with electrical current of different intensity. Electrogustometry is especially useful in estimating the efficiency of sensory pathways. However, if we want to examine taste sensitivity to individual taste categories we should use more laborious gustometry with the application of taste substances, which main advantage is the use of physiological stimuli [57]. Taste impairment may provide a good indicator to the course of some diseases such as diabetes mellitus in which hypogeusia predicts occurrence of degenerative complications. Dysgeusia may induce nutritional disorders and contribute to wasting in chronic liver disease, cancer, or human immunodeficiency virus infected patients. Mechanisms involved in dysgeusia are more than one in a patient. Taste disturbance may be secondary to a variety of causes that include zinc deficiency, lesions of the lingual epithelium, neurological impairment, and adverse events of medication.

### Recommendations

Testing the taste capacity represents a diagnostic tool that is helpful in the clinical discrimination of smell and taste disorder in patients with smell problems complaining of combined loss of smell and taste, and in patients with isolated taste disorders.

## Nasal Nitric Oxide

### Definition

Nitric oxide (NO) is a colourless, odourless gas that is present in air exhaled through the mouth or nose. NO is produced from arginine and oxygen by nitric oxide synthase (NOS). Constitutively expressed neuronal and endothelial forms exist as well as an induced form, iNOS, which appears to be up regulated within the respiratory tract in response to pro-inflammatory signals. NO came to prominence for its role in vasodilatation [58] and subsequently as a neurotransmitter and inflammatory mediator [59]. The role of NO in the airways is complex, possibly including antibacterial effects, pro-inflammatory effects, and regulation of blood flow and ciliary beat frequency. Exhaled NO (eNO) levels are raised in eosinophilic asthma [60] and measurement of this has become a standardised, but not yet widespread, tool in diagnosis and management of asthma. It can potentially provide a rapid, low cost, objective measure of lower airway inflammation.

Far greater levels of NO are produced in the upper than in the lower respiratory tract, with contributions from the sinuses and to a lesser extent from the nasal mucosa [61].

### Objectives

Measurement of nasal NO (nNO) may represent a useful tool for research purposes as well as for sreening for PCD. Nasal nitric oxide may be normal, raised or lowered in disease states; however measurement may be a useful tool in the diagnosis and management of patients with chronic rhinosinusitis, nasal polyps, and CF, as well as in the diagnosis of PCD. Measuring both bronchial and nasal nitric oxide may assist the combined management of upper and lower airways.

### Nasal NO

High levels of NO are produced constitutively in normal individuals within the paranasal sinuses by calcium-independent nitric oxide synthase, with levels measured at 20-25 ppb [62]. Additionally, nitric oxide is also formed in the nasal mucosa by inducible NOS (iNOS) in response to inflammation. NO and its metabolites are toxic to micro-organisms and likely form part of the innate defense mechanism of the respiratory tract. NO may also stimulate cilia beat frequency within the epithelium and regulate nasal vascular tone.

### Technique

As for eNO, nNO can also be measured by chemilluminescence, using non-invasive techniques, providing immediate results. A number of different techniques have been used to ensure sampling from the upper airways only including breath holding and breathing against resistance. Guidelines for measurement have been published [63].

### Sensitivity and specificity

In contrast to measuring eNO, high baseline levels in nNO make background environmental NO levels less of a problem. Conversely, there is a high degree of inter-individual variability amongst healthy controls. Moreover, there is also a significant degree of intra-individual variation over time, meaning that changes of 20-25% or less may be accounted for by normal variation rather than change in disease status or response to medication [64]. Additionally, the lack of universal standardization of testing procedures means levels recorded by different study groups vary considerably even amongst equivalent patient populations. The factors affecting eNO levels such as recent exercise or time of day, may similarly affect nNO measurements. Local factors such as nasal volume and patency may also be important.

### Outcomes

Despite the above limitations nNO has a number of potentially useful clinical applications. With regards to diagnosis, nNO is useful as a screening tool for patients with possible PCD; levels less than 100 ppb, particularly if these persist following decongestion, should stimulate investigation of mucociliary structure and function. The test is objective and may be easier to perform than a saccharine clearance test in younger children. Similarly, nNO may provide a useful tool in diagnosis of CF in the context of upper respiratory tract symptoms; levels significantly lower than in controls have been reported in some studies, but not others. nNO has a potential role in the diagnosis and assessment of CRS, especially when associated with NP. Interestingly, despite the increased expression of iNOS in polyp epithelium [65], low nNO levels have been found in two large studies [66]. Moreover, nNO inversely correlated with endoscopic NP size, CT scores and clinical severity of disease [67]. Conversely, in a study involving chronic rhinosinusitis patients with and without polyps, no correlation between nNO and CT scores was found, although patients were again found to have lower baseline nNO than controls [68].

Low nNO levels in chronic rhinosinusitis are thought to reflect obstruction at the sinus ostium and impairment of gas transfer out from the sinuses. This is supported by the finding of raised nNO following medical and surgical [65] treatment of rhinosinusitis with or without polyps.

A number of recent studies have focused on the possible use of humming to improve the sensitivity of nNO measurements. Weitzberg and Lundberg [69] found that humming induced a large increase in nNO and that these increases were not detected in patients with nasal polyps and sinus ostium obstruction. Furthermore, they suggest that absence of a normal response to humming during nNO measurement could be used to identify allergic rhinitis with sinus ostium obstruction. Whether this adds significant value to standard testing has yet to be fully appreciated.

### Recommendations

Nasal NO is a useful measure to alert the clinician to a possible defect in mucociliary clearance (PCD, CF) and may have in the evaluation of the patency of the sinus ostium Variable baseline levels of nNO and the modest changes which may occur in allergic rhinitis or following treatment make nNO measurement of little value in the diagnosis and management of uncomplicated rhinitis.

## Nasal Sampling: lavages, cytology, biopsies

### Rationale

Inflammation of the nose and sinuses is represented within the nasal mucosa and secretions. A variety of approaches have been used to monitor nasal inflammation to investigate disease processes and to evaluate the effect of therapeutic intervention. These approaches include nasal lavage, different ways to obtain nasal cytology, nasal biopsy, and nasal NO-measurements.

### Objectives

To compare different sampling methods of the nose and indicate the strength and weaknesses.

### Techniques

#### Nasal blown secretions

In this method, secretions in the nasal airways are blown onto wax paper or a plastic wrap and then placed onto a glass slide. Microscopic evaluation allows the discrimination of epithelial cells from granulocytes.

#### Nasal lavage

Nasal lavage is the introduction of fluid into the nasal cavity and its recovery after a predetermined dwell time. Nasal lavage is simple and rapid to perform, is well tolerated, and provides a sample that allows us to evaluate the content of the secretion in the nasal lumen such as protein, cells, mediators and cytokines. A range of techniques has been used to instil and recover fluid from the nasal cavity. Usually a volume of 2.5 mL to 5.0 mL 0.9% NaCl, prewarmed to 37°C, is instilled within each nostril with an 80% recovery (range, 65% to 90%). An agent to disrupt the disulphide bonds of the mucus polypeptide chains can be included. In situations of extreme nasal blockage, the obstruction of the nasal lumen will limit the amount of fluid that can be retained within the nasal cavity, and smaller lavage volumes need to be used under such circumstances. The consistency of the findings in allergic and infective rhinitis for a range of different measures in nasal lavage fluid supports the concept that this method of nasal evaluation provides reliable information of relevance to disease activity although normalization of the variable recovery can be difficult. However, repeated nasal lavage is associated with a significant reduction in histamine concentration. Also there is considerable variability between subjects in eosinophil luminal recruitment and activation.

#### Sinus packs or filter paper

Pre-weighed sinus packs are placed on the floor of the nasal cavity between the septum and inferior turbinate for 5 min and then placed back in a Falcon tube [70]. In order to mobilize the nasal secretions out of the sinus pack, the sinus pack is washed with 3 ml of 0.9% NaCl solution. The sinus pack is then placed into the shaft of a syringe and the sinus pack is squeezed by moving the piston of the syringe. After this first pressure the shaft containing the sinus pack is placed into a Falcon tube and centrifuged at 1,500 g for 10 min to recover all fluid.

If irritation of the nasal mucosa is an issue, thin filter paper that can be inserted without touching the nasal mucosa, can be used instead of sinus packs. The amount of secretion that can be absorbed in this way is however more limited [71,72].

#### Microsuction technique

Nasal secretions can be collected by direct aspiration as has been described by Biewenga [73]. The samples can be collected by repeated aspiration into a pre-weighed plastic sampling tube immediately followed by aspiration of a known volume (1.0 ml) of PBS containing 10% of Mesna. Mesna acts by disrupting the disulphide bonds of the mucus polypeptide chains, and is necessary to obtain a good quality supernatant. The direct aspiration system combines the advantages of minimal irritation of the nasal mucosa with the facility to determine concentrations per gram of secretion.

#### Nasal brush

A small nylon brush used for cell sampling, is introduced in the middle meatus of the nose and turned carefully. The brush is immediately placed in a 5 ml polystyrene plastic tube containing 5 ml of PBS and is cut off just above the bristles. The brush can then be shaken vigorously in the solution and carefully brushed off against the wall of the tube. The tubes are centrifuged at 400 g for 10 minutes [74]. Both the supernatant and cells can be used for analysis. Nasal brush give information on living epithelial cells which is an advantage over nasal lavage, however the sampled area is smaller. Brushing can reliably be used in babies and small children [75].

#### Nasal scraping

Nasal scraping can be performed with the Rhinoprobe [76,77]. The cupped tip of the disposable probe is gently passed over the mucosal surface of the medial aspect of the inferior turbinate. Two or three short scrapes of the epithelial layer are made to obtain a sample. The specimen is spread onto a plain slide and immediately fixed for at least 1 minute in 95% ethyl alcohol. Nasal scrapings give information on living epithelial cells sometimes in larger lumps which is an advantage over nasal lavage, however the area sampled is smaller than lavage and brush.

#### Nasal biopsy specimens

Biopsy specimens can be taken from the nasal mucosa, usually from the inferior turbinate. High quality 2.5-mm biopsy specimens can be taken under direct vision with nasal biopsy forceps, such as Gerritsma forceps (Fokkens' forceps), without visible damage to the epithelium of the sample and with sufficient depth of lamina propria [78]. Local anaesthesia can be achieved by placing a cotton-wool carrier with 50 to 100 mg of cocaine and 3 drops of epinephrine (1:1000) under the inferior turbinate without touching the area from which the biopsy specimen is taken. For light microscopic evaluation, the biopsy specimens are embedded in Tissue-Tek II OCT compound in a gelatin capsule and frozen immediately. Biopsies can be taken a number of times within one patient without causing significant problems [79]. The minimum number of sections required to give a sufficient number of fields to assure acceptable accuracy (5%) was determined to be 2 on the basis of a summation average graph [80].

### Comparison of different techniques

A comparison of the different techniques is shown in Table 5. The choice of the technique depends on the diagnostic or research question asked.

**Table 5:** Comparison of different techniques

Method	Advantage	Disadvantage
Nasal blown secretions	- easy to perform	- subject must be able to blow nose - no information about mucosa

Nasal lavage	- easy to perform - luminal proteins, cells, mediators and cytokines	- reliability depends ability of subject to close nasopharynx - dilution of mediators and cytokines - variable recovery of fluid - no information about mucosa

Sinus packs or filter paper	- no/limited dilution of mediators	- may irritate the nose - cannot collect cells - no information about mucosa - more difficult than lavage

Microsuction technique	- no dilution of mediators	- representative sample? - technically difficult - cannot collect cells - no information about mucosa

Nasal brush	- sample of epithelium	- no sample of deeper layers - no information about nasal lumen - technically more difficult

Nasal scraping	- sample of epithelium	- no sample of deeper layers - no information about nasal lumen - technically more difficult

Nasal NO	- non-invasive	- measure of inflammation and blockage

Nasal biopsy	- sample of total nasal mucosa	- no information about nasal lumen - technically difficult

Data on the comparisons between different techniques are limited, but cells and mediators within nasal lavage have been reported to show some correlation [81].

Cells determined by nasal brush has been shown to be comparative to nasal lavage after nasal allergen provocation [82]. Two studies show moderate to good correlation between cells recovered from biopsy and brush [83], however in one of these studies in which repetitive nasal provocations were given the number of inflammatory cells differed considerably on a day to day base [84].

## Evaluation of Nasal Patency

### Rationale

One of the primary functions of the nose is to humidify, filter and warm the inspired air. A patent nose with lack of anatomic and/or mucosal disease is a prerequisite for the transport of inspired air from the nose to the lower airways. A proper clinical examination of the nose allows the clinician to evaluate the nasal function. Inspection of the nose at rest and during inspiration may show normal anatomy or pathology causing impaired nasal functioning like vestibular stenosis, collapse of the nostrils during inspiration called alar insufficiency, asymmetry in the nostrils or severe nasal septal deviation. Anterior rhinoscopy enables the clinician to distinguish between mucosal and anatomic problems associated with nasal dysfunction. Other clinical tools like nasal endoscopy and posterior rhinoscopy allow experienced clinicians to evaluate the entire endonasal cavity and nasopharynx including the choanal region respectively.

A more objective evaluation of nasal patency may be required in patients suffering from nasal obstruction due to anatomic or mucosal pathology, or in the context of provocation studies and clinical trials. Nasal patency can be assessed by different means, each technique dealing with a different aspect of patency: nasal flow, nasal cross-sectional diameter and nasal resistance during respiration. An overview is provided on the different techniques with technical aspects, advantages and disadvantages of every technique and recommendations for use in practice.

### Objectives

Nasal obstruction is often reported by patients with anatomic or mucosal nasal disease and can be scored subjectively on a visual analogue (VAS) scale. Objective evaluation tools of nasal obstruction are often warranted in patients suffering from nasal obstruction or for evaluation of mucosal changes in provocation studies or clinical trials.

Nasal patency can be monitored objectively by measuring the following parameters:

1/nasal air flow passing through the nose during nasal respiration, evaluated with the nasal peak inspiratory and expiratory flow (PNIF and PNEF)

2/the volume of the nasal cavity evaluated with acoustic rhinometry and

3/the nasal airflow and pressure during nasal respiration evaluated with rhinomanometry.

Differences exist in indication for clinical use of each technique, the interpretation of the results, the validation of the technique, and the cost of the equipment.

### Definitions

#### 1/Peak nasal inspiratory flow (PNIF)

**Figure 12:**
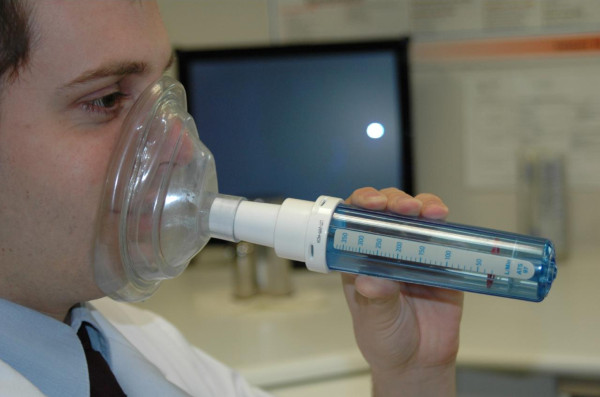
**Peak nasal inspiratory flow measurement**.

Nasal peak flow evaluation (Figure 12) represents a physiologic measure of the air flow through both nasal cavities during forced inspiration and/or expiration expressed in liter per minute. The PNIF is the best validated technique for the evaluation of nasal flow through the nose. Nasal inspiration correlates most with the subjective feeling of obstruction and is the best validated technique for monitoring nasal flow in clinical trials and after nasal provocation. In contrast to PNIF, PNEF is less validated and used in clinical practice in view of the mucus being blown into the peak flow meter and subjective discomfort during maximal expiration in patients with mucosal disease [85].

#### 2/Rhinomanometry

**Figure 13:**
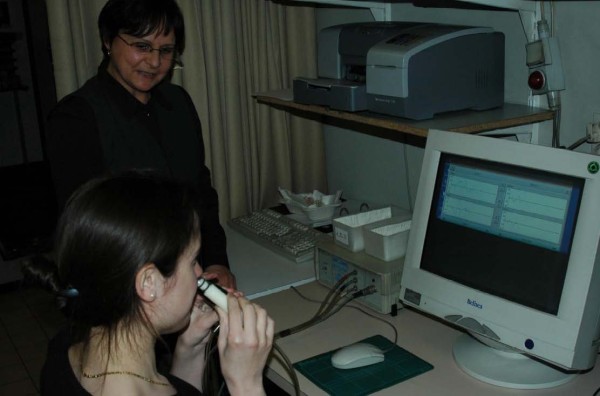
**Active anterior rhinomanometry**.

Active anterior rhinomanometry (Figure 13) represents a physiologic measure of nasal air flow and pressure during normal inspiration and expiration. It is considered the standard technique for the evaluation of nasal airflow resistance, hence providing a functional measure of nasal patency. Depending on the position of the probes for registration, anterior or posterior rhinomanometry can be performed, both being valid techniques. When the probe is placed in the mouth, posterior rhinomanometry values can be obtained for both nasal cavities together or for one nasal cavity when sealing of one nostril. In anterior rhinomanometry, the pressure-sensing tube is placed in one nostril and data represent unilateral pressure and flow measures. The anterior rhinomanometry is often recommended for its' ease of use.

#### 3/Acoustic rhinometry

**Figure 14:**
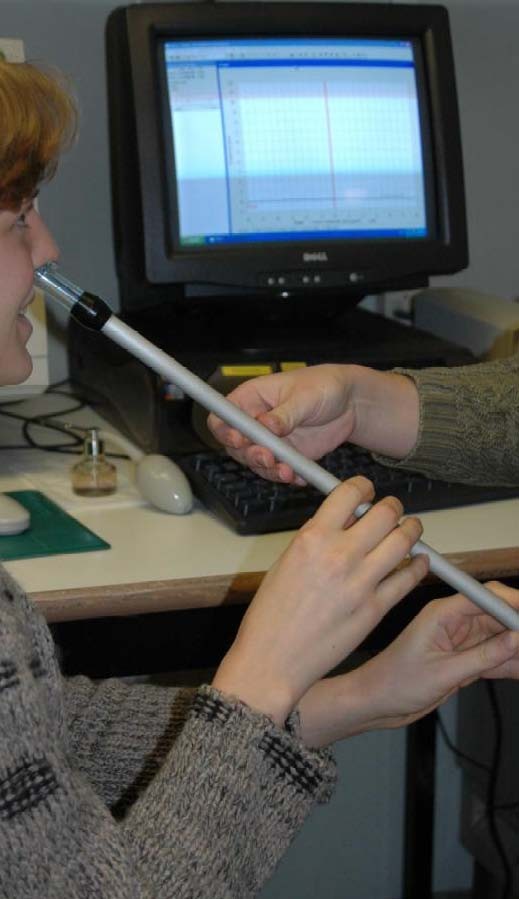
**Acoustic rhinometry**.

Acoustic rhinometry is a non-physiologic measure of nasal patency, measuring echoes of sound impulses sent into one nostril (Figure 14). The measurement provides information on the nasal luminal anatomic structures, either as a measure of nasal volume over a standard distance into the nostril or as the minimal cross-sectional area within the nasal cavity. The measurement is performed in each nasal cavity separately.

### Techniques

#### 1/Peak nasal inspiratory flow

##### Description

The PNIF meter consists of a device evaluating the airflow in liter per minute passing through the tube, and an anaesthesia mask sealing the nose and mouth in an airtight way during nasal inspiration (Figure 12). The anaesthesia mask should have the proper seize, i.e. not too large preventing leakage of air, or too small compressing the nose with impairment of nasal inspiration.

The patient is asked to blow the nose first. Then he is instructed to exhale maximally, after which the mask is placed over the nose and mouth with airtight seal around the mouth and nose. The patient is then asked to inspire forcefully through the nose with closed lips. Lip closure is verified during the PNIF test through inspection of lip closure through the transparent anesthesia mask in order to prevent the generation of false high values.

The nasal flow is expressed in liter per minute, and consecutive measurements are performed. Taking the best of 3 outcomes with less than 10% variation is considered to be the best means of expression of the result [85].

##### Devices

Youlten peak flow meter (Clement Clark International) attached to anesthesia mask.

##### Advantages

- cheap and portable equipment

- assistance not required after short training session (5 minutes)

- rapid and easy to use

- good correlation with subjective feeling of nasal obstruction

##### Disadvantages

- influence of lower airway function

- cooperation of patient required

- no unilateral measurement possible

- impossible in patients with alar collapse during inspiration

#### 2/Anterior rhinomanometry

##### Description

Rhinomanometry provides a quantitative measure of nasal airway resistance. In anterior rhinomanometry (Figure 13), the patient is asked to blow his nose, sits in an upright position and the pressure sensing tube is placed in one nostril with total seal by taping off the nostril or inserting an olive-like device or nozzle. The contra-lateral nostril is sealed with either tape or olive-like device blocking off nostril. Unilateral measurements are being performed, demonstrating any asymmetry or abnormality in nasal airway resistance.

When the measurements are performed before and after the application of a nasal decongestant spray, the differences in resistance can be attributed to nasal mucosal congestion. Data obtained after nasal decongestion allow the evaluation of anatomical factors influencing resistance.

##### Advantages

- specific measurement of nasal resistance

- information on each nostril separately

- relatively ease technique

- not time-consuming

##### Disadvantages

- relatively expensive equipment

- equipment not portable

- operator required

- impossible in case of total obstruction of one nostril

- interference with nasal cycle

- weak correlation with subjective nasal congestion

#### 3/Acoustic rhinometry

##### Description

The acoustic rhinometer generates an acoustic wave that is transmitted through a tube into one nostril (Figure 14). The size and the pattern of the reflected sound waves provide information on the structure and dimensions of the nasal cavity, with the time delay of reflections correlating with the distance from the nostril. The conversion of echo measurements to nasal volume requires mathematical calculations and theoretical assumptions.

The patient sits in upright position, clears its' nose and places the nosepiece into the nostril. The nosepiece should fit the nostril, ensuring an airtight seal. Measurements are performed during breath holding.

When the measurements are performed before and after the application of a nasal decongestant spray, the differences in cross-sectional diameter of the nose can be attributed to nasal mucosal congestion. Data obtained after nasal decongestion allow the evaluation of anatomical factors influencing the cross-sectional diameter of the nose.

##### Advantages

- easy to use

- minimal patient cooperation

- information of each nostril separately

##### Disadvantages

- non-physiological measure of nasal patency

- operator required

- interference with nasal cycle

- weak correlation with subjective nasal congestion

##### Other techniques

##### * Anterior and posterior rhinoscopy

Anterior rhinoscopy allows the examiner to evaluate the anterior half of the nasal cavity, discriminating mucosal from anatomic disease. The subjective evaluation of nasal patency by the appreciation of the endonasal lumen, anatomic relationships and mucosal disease is the most important diagnostic tool for the evaluation of nasal patency.

In posterior rhinoscopy, a small mirror is placed in the oropharynx behind the soft palate, allowing the evaluation of the choanal openings and mucosal disease at the nasopharynx. In experienced hands, this technique may be helpful in the diagnosis of posterior nasal disease but its' routine use is hampered by a vomiting reflex in a substantial portion of patients. As nasal endoscopy allows the full appreciation of the nasal cavity and nasopharynx, the diagnostic role of posterior rhinoscopy has become restricted to those cases where nasal endoscopy is not available or desired like in young children.

##### * Nasal endoscopy (Figure 8)

A rigid nasal endoscope with 0° or 30° angle is gently inserted into each nostril, examining the inferior and middle meatus, nasopharynx, septal anatomy and endonasal mucosal state. Due to the limited discomfort for the patient, the possibility of thorough examination of the nasal cavity and the information on the mucosal condition, nasal endoscopy is the gold standard for evaluation of nose in patients with sinonasal disease [2].

##### * Mirror test (Figure 7)

Holding a cold metal spatula or plate under the nose during expiration allows the examiner to evaluation the condensation of exhaled air onto the metal device. Specific attention is being paid to the symmetry of the condensation or unilateral absence of condensation. In view of the non-invasive character of the test, the rapid, cheap and easy methodology, it can be useful as a screening tool for evaluation of nasal patency in children. The fact that the expiratory flow does not correlate well with the subjective feeling of nasal obstruction makes this test of limited diagnostic value in the evaluation of nasal congestion.

### Variability and correlation with subjective symptoms

#### 1/Peak nasal inspiratory flow

##### * Variability

- PNIF measures may vary from the characteristics of the flow meter and mask used for analysis.

- Intra-individual variations may relate to diurnal changes, with PNIF values being lower in the morning and highest at dinner [85].

- Inter-individual variations may relate to subject technique, respiratory function, and cooperation. Hormonal changes, microbial and environmental factors affecting the nasal mucosal congestion like temperature and smoke, may cause changes in PNIF between individuals.

##### * Correlation between PNIF and nasal obstruction symptom

A strong positive correlation has been reported between PNIF and the subjective feeling of nasal obstruction determined by means of questionnaires [86-88]. This positive correlation was not consistently found in all studies [89,90].

#### 2/Rhinomanometry

##### * Variability

The nasal cycle interferes with the measurement of cross-sectional diameter and data should be interpreted in this respect.

##### * Correlation between rhinomanometry and nasal obstruction

Ojbective measures of nasal resistance do not correlate well with subjective syptoms. Some studies however show corresponding results between rhinomanometry and subjective symptom scoring after inducing (de)congestion [91,92].

#### 3/Acoustic rhinometry

##### * Variability

- The nasal cycle interferes with the measurement of cross-sectional diameter and data should be interpreted in this respect.

- Large inter-individual variations are present.

##### * Correlation between acoustic rhinometry data and nasal obstruction

In healthy individuals, there is poor correlation between acoustic rhinometry data and subjective nasal obstruction scores, whereas correlations are better in congested subjects [93,94].

### Recommendations

Depending on the specific aim of nasal patency and flow evaluation, one may rely on different tools for the evaluation of nasal patency and flow. A table with clinical use and indicitions is provided below (Table 6).

**Table 6:** Tools for the evaluation of nasal patency and flow

	PNIF	Rhinomanometry	Acoustic rhinometry
Diagnostic purposes			
- unilateral disease	-	++	++
- correlation with syptoms	+++	+	+

Use in children			
2-6 y	-	+	+++
6-18 y	-	++	+++

Provocation studies	+++	+++	+++

Clinical trials	+++	+++	+++

Home monitoring	+++	-	-

Evaluation of effect of treatment	+++	+++	+++

## Microbiology

### Rationale

The evaluation of the presence of virulent bacteria inside the nasal and sinus cavities represents a diagnostic tool in rhinosinusitis. Although there is no evidence for benefit in establishing diagnosis or improving treatment outcomes by using routine microbiological analysis of nasal or sinus samples in uncomplicated acute or chronic rhinitis or rhinosinusitis [2], research which was focused during the past decade on the role of bacterial superantigens, biofilms, response to fungal antigens, osteitis and intracellular bacterial growth in nasal and sinus mucosa may give rise to broader microbiological analysis of samples from nose and sinuses, involving new, more sensitive detection techniques. Although detection of microbes or their products in the samples is highly improved, problems remain with establishing relevant microbial pathogenicity, virulence, viability on one hand, and relevance of the detected microbes to the development of symptoms/disease on the other hand.

### Colonization versus infection

Rhinosinusitis is defined as inflammation of the nose and sinuses and the diagnosis is based on characteristic symptoms. The definition does not imply infection as the etiological cause. The nasal and sinus cavities and nasopharynx are colonized with commensal bacteria, but also (especially in children) with those belonging to usually pathogenic strains (like Staphyloccus aureus in adults; Streptococcus pneumoniae or Haemophilus influenzae in children) [95]. Such colonization does not lead to marked inflammatory cell activation or symptoms.

In symptomatic patients with clinical evidence of infectious rhinosinusitis, the identified pathogen may be considered as the cause if it is present in more than 1000 colony forming units (cfu) per ml (usually more than 10.000 cfu. in acute rhinosinusitis), and there is an inflammatory response of the host, proved by increased number of leukocytes in the samples [96].

### Objectives

- to establish indications for microbiological assessment of nasal or sinus samples in non-complicated and complicated rhinitis/rhinosinusitis

- to define advantages and disadvantages of conventional culture-based phenotyping of bacteria and monitoring antimicrobial sensitivity, serologic response measurements and molecular techniques based on detection of proteins or nucleic acid directly or by amplification

- to find an evidence-based algorithm using different microbiological sampling and detection techniques, coupled with monitoring of the host response, and adequate interpretation of the results in terms of distinguishing between colonization and infection, relevance of microbial pathogenicity and virulence for the development of symptoms and impact of detection of microbes and their antimicrobial sensitivity on treatment.

### Technique/Instrumentation

Nasal and sinus samples for microbiological assessment are taken as swabs, aspirates, lavages or biopsies. Monitoring of the local host response may be done using cytology, biopsy or lavage, or systemically using serology. The poor correlation between nasal/nasopharyngeal and sinus swabs suggests that sinus sample contamination with nasal or oral cavity colonizing bacteria may lead to misinterpretation of the microbiological results. To obtain adequate samples, disinfection of the vestibule is indicated if the sinus swab or lavage is taken via nasal endoscopy or sinus puncture. Maxillary sinus samples can be taken through inferior meatal puncture, transoral puncture or endoscopically guided through the middle meatus. Correlation of endoscopically taken samples from maxillary sinuses, compared to maxillary sinus puncture is high in most of the studies [97].

Routine bacteriological analyses of the samples are based on cultivation on selective plates and phenotyping and identification of gram positive, gram negative and anaerobic bacteria. Processing and the time elapsed from taking the samples to cultivating has an impact on detection sensitivity, at least for some bacterial strains (especially anaerobes). Cultivating is successful in detecting only viable bacteria and counting the colony forming units is relevant for defining significance of bacterial growth to symptoms. Different methods of antimicrobial sensitivity testing may be applied.

For the detection of intracellular bacteria, immunohistochemistry may demonstrate a specific bacterial strain in mucosal tissue. Detection and amplification of microbial RNA and DNA has improved detection sensitivity, but does not give information on microbial viability. Real-time quantitative polymerase chain reaction (RT-PCR) may give information of the number of bacteria, but sequential samples are needed to prove viability. For the detection of bacteria in biofilm [98], fluorescent in situ hybridization (FISH) is usually applied, coupled with confocal microscopy.

### Sensitivity and specificity

The recovery rate in the samples of chronic maxillary sinusitis, both aerobic and anaerobic bacteria, varied in different studies from 45% to 92%, and is dependent on adequate sampling, culture techniques and detecting techniques. Although sinonasal bacteria are detected in up to 90% of chronic rhinosinusitis patients, their role in severity and pattern of inflammation is even less clear than in ARS. In a study where bacteria were cultivated in 88% of the sinus samples from patients operated for CRS, inflammation was confirmed microscopically in only 11% [99]. PCR may detect minute amounts of bacterial DNA, which may suggest extremely high sensitivity but gives no information on bacterial viability, or impact on the inflammation. Quantitative RT-PCR may offer information on bacterial count from small samples, but its specificity and sensitivity depends on the primers used for analysis. In the very few sinusitis studies comparing PCR with conventional microbiology, PCR was not found to be more sensitive or specific than cultivation techniques [100].

### Outcomes

There is no evidence that microbiological assessment of nasal or sinus samples has any impact on outcomes in rhinitis/rhinosinusitis. Although randomized double blind placebo controlled trials indicate antibiotic treatment of ARS is significantly superior to placebo [2], there is no evidence that antibiotic treatment based on microbiological sampling gives better outcomes compared to empiric antimicrobial treatment in non-complicated acute rhinosinusitis. Thus identification of pathogens in ARS is not indicated. European guidelines for the treatment of ARS suggest that ARS non-responsive to empirical antimicrobial treatment and topical nasal steroids, as well as complicated ARS, should be referred to an ENT specialist. At that time, further diagnostic procedures including microbiology are advised [2].

### Recommendation

Microbiological assessment is not to be used routinely in diagnosis of rhinitis/rhinosinusitis. ARS non-responsive to empirical antimicrobial treatment and topical nasal steroids should be referred to an ENT specialist, where further diagnostic procedures, including microbiology, should be done.

## Evaluation of Mucociliary Clearance

### Rationale

In children with rhinosinusitis presenting with longstanding and persistent anterior rhinorrhoea, one may be interested in the evaluation of the function of the mucociliary clearance system for diagnostic purposes [101]. By their coordinated movement, the ciliae lining the respiratory epithelium transport the mucus layer with entrapped inhaled particles from the nasal cavity towards the hypopharynx [102]. In this way, about 10 ml of mucus is transported daily from the upper airways towards the hypopharynx, ultimately being swallowed and cleared from the airways. Normal mucociliary transport is essential for the maintenance of healthy sinuses. In case of infection and/or congential dysfunction of the ciliae like in primary ciliary dyskinesia (PCD) [101], the mucociliary transport is inadequately or not taken place. In PCD, lack of mucociliary transport may lead to chronic rhinosinusitis and bronchiectasis. In chronic inflammation, mucostasis, hypoxia, microbial products, toxic inflammatory mediators may induce secondary ciliary changes, i.e. secondary ciliary dyskinesia (SCD), with inadaquate mucociliary transport.

### Definition

Objective evaluation of the mucociliary clearance of the upper airways in order to quantify the proper function of the ciliae of the respiratory epithelium lining the upper respiratory tract.

### Techniques

#### Mucociliary clearance time

The mucociliary transport (MCT) mechanism ensures the clearance of entrapped particles in the mucus lining the nasal mucosa towards the hypopharynx. Several non-absorbable substances have been used for the evaluation of MCT in patients.

The saccharine test evaluates the time a patient needs to have a sweet taste after placement of a 1-2 mm particle of saccharine on the inferior turbinate mucosa 1 cm from the anterior end. The patient has to sit quitely with the head bent forward and without sniffing, coughing, sneezing, drinking or eating during the investigation.

Alternatively, one can monitor the time needed for a dye like methylene blue to be transported from the mucosa of the anterior third of the nasal cavity towards the hypopharynx. Other substances like technetium-99m-labeled iron oxide have also been used. The MCT is considered to be normal below 15 minutes, and should be less than 1 hour.

As the MCT can only be measured in cooperative patients with patent nasal cavities and in the absence of severe mucosal disease, this test has limited diagnostic value due to its low sensitivity and specificity. Furthermore, the test takes a long time and has a high incidence of false positive and negative results [101].

#### Electron microscopy

Harvesting epithelial cells is performed by scraping along the inferior and middle turbinates by the use of a sterile cytology brush. These epithelial cells can be used for either structural investigation of the cilia of nasal epithelial cells with electron microscopy or for measuring ciliary beat frequency [103].

In primary and secondary ciliary dysfunction, several abnormalities can be observed in the dynein structures of the epithelial ciliae like total/partial absence of dynein arms, aberrant organization of the dynein arms and/or disorientation [101]. PCD is associated with the latter abnormalities but SCD may also present with these structural abnormalities. Therefore, electron microscopic evaluation of harvested epithelial cells may aid in the diagnosis of PCD, but is not 100% sensitive nor specific.

#### Ciliary beat frequency measurement

Harvested epithelial cells can be evaluated for ciliary beat frequency (CBF) and the ciliary wave form analyzed in detail by digital high speed video imaging. The evaluation of the frequency of the beating of cilia as well as the evaluation of their coordinated movement can be performed by computerized programs using a Fast Fourrier analysis. Normal values of CBF vary upon the methodology used, the age of the patient, and the culture conditions. The demonstration of normal CBF and beat pattern excludes the diagnosis of PCD.

#### Ciliogenesis in vitro

The evaluation of ciliogenesis *in vitro *constitutes the gold standard for diagnosis of PCD, allowing the differentiation between primary and secondary ciliary dyskinesia. A biopsy of the nasal mucosa is taken, and nasal epithelial cells are dissociated by enzymatic digestion and incubated for 6 to 8 weeks until cilia reappear on the apical side of the epithelial cells. The new cilia can be evaluated for their electron microscopic structure and coordinated activity. In PCD patients, no ciliogenesis takes place whereas patients with ciliary dysfunction due to infection/inflammation present with properly functioning ciliae after ciliogenesis.

### Recommendations

No ideal test is available for the diagnosis of PCD. In case of suspicion of PCD in a patient with rhinosinusitis since birth, familial history of PCD and associated features of Kartagener syndrome, i.e. situs inversus and infertility, one should consider diagnostic tests of ciliary function by evaluation of CBF, electron microscopic evaluation of the dynein arms of the cilia, and/or evaluation of the cilia after ciliogenesis *in vitro*. As these techniques are not available in routine ENT practice, one may rely on measuring nasal NO levels as low NO levels have been associated with PCD and therefore represent a good screening tool for PCD (see chapter on NO measurement).

## Blood and Additional Tests

### Rationale

Blood analysis may confirm or refute the definite diagnosis in specific clinical conditions.

### Rhinitis

Blood analyses including tests for allergen-specific IgE have been dealt with in the section on allergy testing. In severe non-infectious, non-allergic rhinitis, one may consider full blood count, including eosinophils, thyroid function, thyroid auto- antibodies, anti- nuclear antibodies, extractable nuclear antibodies (anti- Ro and anti-La are usually positive in Sjogren's syndrome), pregnancy test or tests for drugs of addiction on urine. Sjogren's syndrome (SjS) is a relatively common autoimmune disease characterized by oral and ocular dryness. Patients may present to the rhinitis clinic with symptoms of nasal obstruction, dryness or cough. There is an increasing need for simple, sensitive and rapid technologies for the diagnosis of SjS and other autoimmune diseases. A quick version of luciferase immunoprecipitation systems (QLIPS) can now be used to produce a rapid, specific and quantitative test to detect auto-antibodies associated with SjS. Ro52 was the most informative with antibody titers in the Ro52-seropositive SjS samples approximately 1000 times higher than in healthy controls. Validation of the anti-Ro52 QLIPS test showed 66% sensitivity and 100% specificity and has the potential to be adapted for point-of-care evaluation of patients with SjS and other rheumatologic diseases [104].The need for biopsy looking for lymphocytic infiltration of salivary glands should be reduced and in any case is inaccurate in elderly patients [105].

In case of rhinorrhoea only, especially if unilateral, beta2-transferrin should be measured in nasal secretions. If present, the secretions are cerebro-spinal fluid and reveal a skull base defect. Beta-2 transferrin is a carbohydrate-free (desialated) isoform of transferrin, which is almost exclusively found in the CSF- blood or nasal secretion does not disturb the test. Beta-2 transferrin is not present in blood, nasal mucus, tears or mucosal discharge. This protein was first described in 1979. Intense research over the last decade has validated its characteristics and value in clinical use as a specific CSF marker [106]. Beta-2 transferrin was reported to have a sensitivity of near 100% and a specificity of about 95% in a large retrospective study. Detection of glucose in the nasal sample fluid using Glucostix strips has been a traditional method for detection of the presence of CSF in nasal and ear discharge. Glucose detection using Glucostix test strips is not recommended as a confirmatory test due to its lack of specificity and sensitivity. Interpretation of the results is confounded by various factors such as contamination from glucose-containing fluid (tears, nasal mucus, blood in nasal mucus) or relatively low CSF glucose levels (meningitis). Studies have shown that glucose can be detected in airways secretions from people with diabetes mellitus, stress hyperglycaemia and viral colds.

### Rhinosinusitis without nasal polyps

Depending on the clinical history and examination, consider the following analyses:

* full blood count including differential white cell count, ESR and/or C Reactive Protein,

* evaluation of renal, liver and thyroid function

* humoral immunity markers: immunoglobulins, IgG subclasses, specific antibody levels to tetanus, haemophilus, pneumococcus and response to immunization if low,

* cellular immunity markers: T and B cell numbers and ratios

* HIV status.

* Angiotensin converting enzyme (ACE) is usually up-regulated in macrophage activation in diseases such as sarcoidosis and tuberculosis. Of note, the following diagnostic tests aided to diagnose ocular sarcoidosis [107]: negative tuberculin skin test in a BCG-vaccinated patient or in a patient having had a positive tuberculin skin test previously, elevated serum angiotensin converting enzyme (ACE) levels and/or elevated serum lysozyme, chest x-ray revealing bilateral hilar lymphadenopathy (BHL), abnormal liver enzyme tests, and chest CT scan in patients with a negative chest x-ray result.

* c-ANCAs (anti- neutrophil cytoplasmic antibodies) are raised in Wegener's granulomatosis, in 60% of patients where upper respiratory tract alone is involved.

### Rhinosinusitis with nasal polyps

Some specific pathologic entities should be considered in severe nasal polyp disease and require additional investigations.

#### 1/Churg Strauss syndrome(CSS)

ANCAs are present in approximately 40% of patients with CSS. A pANCA pattern with specificity for MPO is found in most ANCA-positive patients. ANCA positivity is mainly associated with glomerular and alveolar capillaritis [108].

Antineutrophil cytoplasmic antibodies (ANCA) are predominantly IgG autoantibodies directed against constituents of primary lysosome granules of neutrophils and monocytes. Several antigenic targets exist: those ANCA directed to proteinase 3 or myeloperoxidase are clinically relevan. The importance of other ANCA remains unknown. Both are strongly associated with small vessel vasculitides, including Wegener's granulomatosis, microscopic polyangiitis, and Churg-Strauss syndrome, and the localised forms of these diseases (eg, pauci-immune necrotising and crescentic glomerulonephritis). ANCA is a useful serological test to assist in diagnosis of small-vessel vasculitides. 85-95% of patients with Wegener's granulomatosis, microscopic polyangiitis, and pauci-immune necrotising and crescentic glomerulonephritis have serum ANCA [109]. Besides their diagnostic potential, ANCA might be valuable in disease monitoring, although the ESR is quicker.

Recent data seem to confirm the long-disputed pathogenic role of these antibodies: myeloperoxidase-ANCA are directly involved in the pathogenesis of necrotizing vasculitis. This is less clear for proteinase 3-ANCA, markers for Wegener's granulomatosis. Complementary proteinase 3, a peptide translated from the antisense DNA strand of proteinase 3 and homologous to several microbial peptides, may be involved in induction of proteinase 3-antineutrophil cytoplasmic autoantibodies.

A strategy based on screening for ANCA with ELISA or fluoroenzymeimmunoassay (FEIA) without prior indirect immunofluorescence (IIF) is a valuable alternative to screening with IIF and confirming with ELISA or FEIA.

Cocaine-induced midline destructive lesions unfortunately have a high prevalence of cytoplasmic antineutrophil cytoplasmic antibodies, limiting this test's usefulness in distinguishing this disorder from Wegener's granulomatosis [110].

#### 2/Aspirin sensitivity

The aspirin provocation test, considered to be the gold standard in the diagnosis of aspirin sensitivity, may be associated with severe adverse reactions; thus, alternative procedures with a higher safety profile are highly desirable. Although the cellular antigen stimulation test (CAST) has been proposed as an alternative a recent study using CAST to measure cys LTs pre and post challenge showed that although the leukocytes of patients with aspirin sensitivity produce higher amounts of Cys-LTs as measured by CAST the assay had a sensitivity of 25%, a specificity of 92.3%, and positive and negative predictive values of 28.7% and 90.7%, respectively. The low sensitivity and predictive values limit the clinical usefulness of this test in the diagnosis of aspirin sensitivity [111].

#### 3/Fungal sinusitis

Fungal spores are continuously inhaled and deposited on the airway mucosa, both in healthy persons as well as in patients with CRS. Five forms of fungal disease affecting the nose and paranasal sinuses have been recognized [112]:

(1) acute invasive fungal rhinosinusitis (including rhinocerebral mucormycosis),

(2) chronic invasive fungal rhinosinusitis,

(3) granulomatous invasive fungal rhinosinusitis,

(4) fungal ball (mycetoma), and

(5) non-invasive (allergic) fungal rhinosinusitis.

There are several potential deficits in the innate and potentially also acquired immunity of CRS patients that might reduce or change their ability to react to fungi. There are not many arguments to suggest a causative role for fungi in CRS with or without nasal polyps. However, due to the intrinsic or induced change in immunity of CRS patients, fungi might have a disease-modifying role.

#### 4/Primary ciliary dyskinesia

Congenital dysfunction of the mucociliary transport system, called primary ciliary dyskinesia (PCD) is a rare heriditary condition, associated with dextrocardia and infertiliy. The discrimination between PCD and secondary dysfunction of the cilia due to microbial or environmental agents, is crucial for the diagnosis. Several diagnostic tools are available. Determination of the mucociliary clearance time is a non-invasive, cheap and rapid diagnostic tool, with relatively low sensitivity and specificity. Nasal NO is reported to be very low in patients with PCD [113] in contrast to CRS, NP and other nasal inflammatory conditions. Therefore, nasal NO measurement represents a valuable screening tool for PCD. Electron microscopic evaluation of the epithelial cilia may provide additional hints for the diagnosis without being 100% specific or sensitive [114]. The definite proof of PCD comes from epithelial cell cultures and of ciliogenesis in vitro [115]. However, the time-consuming and costly nature of this investigation limits its' use as a screening tool.

#### 5/Cystic Fibrosis (CF)

The diagnosis of CF is suspected in case of severe CRS with NP and thickened secretions, hypoplasia of the paranasal sinuses, in association with recurrent broncho-pulmonary infections. CF is an autosomal recessive disease caused by mutations in the the CF transmembrane conductance regulator (CFTR) gene that results in abnormal viscous mucoid secretions in multiple organs and with rhinosinusitis as one of the clinical features beside endobronchial infections and pancreatic insufficiency.

Blood analysis for CFTR gene mutations may demonstrate homozygote and heterozygote gene mutations in a subgroup of CF patients [116]. CT scans of the paranasal sinuses may show features associated with CF: hypoplasia of the frontal and/or sphenoidal sinuses, full opacification of most sinonasal cavities, and pseudomucocoeles in the maxillary sinuses with medialization of the lateral nasal wall.

The sweat test remains the gold standard for diagnosis of CF, as it is non-invasive, cheap and painless, with high sensitivity and specificity.

### Recommendations

* Blood analysis may be an alternative for skin prick test in patients suspect of allergic rhinitis.

* Specific patients with non-allergic, non-infectious rhinitis may require the following analyses: full blood count, including eosinophils, thyroid function, thyroid auto-antibodies, anti- nuclear antibodies, extractable nuclear antibodies in Sjogren's syndrome, pregnancy test or tests for drugs of addiction on urine.

* In specific cases of rhinosinusitis without NP the following analyses are recommended:

* full blood count including differential white cell count, ESR and/or C Reactive Protein,

* evaluation of renal, liver and thyroid function

* humoral immunity markers: immunoglobulins, IgG subclasses, specific antibody levels to tetanus, haemophilus, pneumococcus and response to immunization if low,

* cellular immunity markers: T and B cell numbers and ratios

* HIV status.

* Angiotensin converting enzyme (ACE)

* ANCA

## Imaging in Rhinology

### Rationale

Imaging of the nose and sino-nasal cavity is used as an objective diagnostic tool in establishing the diagnosis and in staging the severity of rhinosinusitis (RS) and nasal polyposis (NP). The diagnosis of RS with/without NP is based on the presence of characteristic clinical symptoms, which are confirmed by either nasal endoscopy or radiographic imaging [2]. Computerized tomography (CT) scans provide substantial information about paranasal sinus anatomy and are mandatory for safe endoscopic sinus surgery. Unlike standard X-ray and ultrasonography (USG), CT scans of the sino-nasal cavity and magnetic resonance imaging [117] provide objective information on the extent of sinus disease and are the most frequently used objective tools in staging of severity of the disease (with the exception of endoscopic staging of polyp size). For evaluation of the bony anatomy and discrimination of the sino-nasal cavities.

### Objectives

The aims of radiologic imaging are the demonstration of the source of individual sino-nasal symptoms, the extent of the sinonasal disease, the relation of the sinonasal problem with surrounding structures and the evaluation of the sinonasal anatomy prior to sinus surgery.

### Techniques

**Figure 15:**
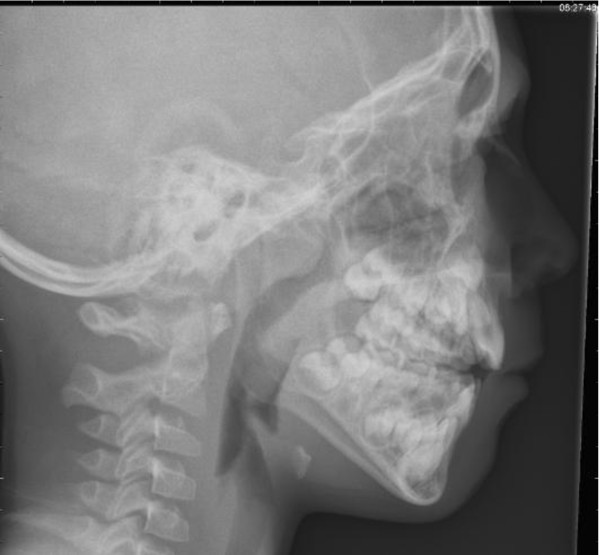
**Plain X ray of skull allowing the evaluation of the adenoid volume in relation to the nasopharyngeal airway passage**.

Plain film radiographs in standard projections (Caldwell and Waters frontal views and Rhese oblique view) provide little information on disease extent and no information on sinus anatomy. They do provide some information on the size of the sinuses and air content in the maxillary and frontal sinuses, but discriminate poorly between bone, mucosa and secretion compared to CT or MRI and may be misleading in diagnosis, and dangerous in surgery. Therefore, plain X-ray radiographs are not advised in routine rhinology clinic. In children with clinical suspicion of adenoid hypertrophy being responsible for nasal obstruction, lateral plain X-ray images (Figure 15) may show the adenoid hypertrophy and be of help in the therapeutic approach in these children.

Following the introduction of CT scans in the 1970s and the concepts of functional endoscopic sinus surgery (FESS) in the 1980s, CT scanning has become the most important imaging modality and helped the development of endoscopic surgery of the sinuses and skull base. The new multi-detector CT (MDCT or MSCT) scanners (4th generation CT scanners) have achieved very short radiation exposure time, taking scans in one cycle, using spiral mode, instead of two projections used in previous generations, which was more time consuming, and uncomfortable for some patients. They also enable multi-planar reconstructions, even in curved planes, like a reconstruction of orthopantomography of the upper jaw and allow precise coronal, axial, sagittal and various 3D reconstructions as well as virtual endoscopy. The MDCT scanning should be done at 3 mm contiguous slices or less throughout the 3 planar scans using both wide («bone») and narrow («soft-tissue») window settings. Such reconstructions are useful in surgical planning, but are not needed for staging. Low-dose protocols should be applied, taking into account the potential pathology. Although new software packages enable 3D reconstructions and virtual endoscopy, besides being time consuming, such images cannot replace surgeons' preoperative analysis of the scans in 3 projections.

**Figure 16:**
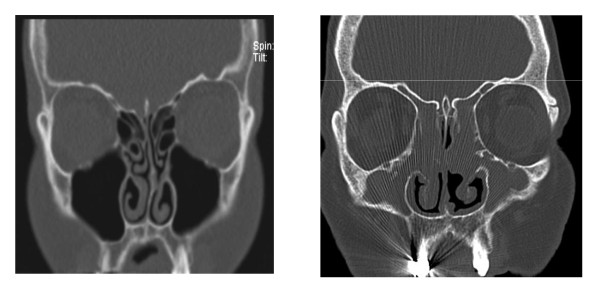
**Coronal CT scan images of normal (left) and NP patient (right)**.

Coronal sections have been the most requested plane on CT imaging of the nose and sinuses as this closest resembles the surgical anatomy encountered in endoscopic sinus surgery, presenting ostio-meatal complex (unit) and relationship between sinuses, orbit, and skull base (Figure 16). Axial sections may be required to visualize the anterior and posterior walls of the sinuses. Reconstruction of MSCT scans in coronal, axial and sagittal planes, enable excellent surgical planning nowadays.

Although more time consuming and costly, the use of MRI is recommended in patients with complicated inflammatory sinus disease extending beyond the boundaries of the sinonasal cavities and/or in patients with suspected neoplasms. T1, T2 and STIR protocols are used, enabling superior resolution to CT on the interfaces between mucosal lining, dura, fat and secretions, but CT and MR present complementary techniques for imaging in these unique circumstances. Contrast-enhanced high resolution SE axial and coronal T1-WI further differentiate soft tissue structures and intracranial and/or intra-orbital extension of pathology can be better demonstrated in this manner. Although there is no ionizing radiation, allowing MRI to be repeated, certain contraindications for its use should be considered such as metal objects, prosthesis and implants, e.g. cardiac pacemaker, implanted cardiac defibrillator, aneurysm clips, carotid artery vascular clamp, neurostimulator, insulin or infusion pump, implanted drug infusion device, bone growth/fusion stimulator, cochlear, otologic, or ear implant. The duration and methodology of MRI can cause claustrophobia.

Ultrasonography of the paranasal sinuses is easily available, cheap and quick, with no irradiation or discomfort involved. Ultrasonography offers an opportunity for repetitive examination, which might be important for the follow-up in evolution of acute rhinosinusitis. Sensitivities have been reported from 29% to 100% and specificities from 55% to 99%, but limited to analysis of maxillary and frontal sinuses. However, it provides little information on disease extent.

### Sensitivity and specificity

The diagnosis of rhinosinusitis is largely based on the patient's history, with radiologic imaging confirming the clinical diagnosis of RS. It is impossible to differentiate between acute and chronic rhinosinusitis based on imaging. CT scan is not indicated as a diagnostic procedure in acute rhinosinusitis, except when a complication is suspected or recurrent rhinosinusitis is not responsive to treatment. As CT and MRI are sensitive enough to detect changes in the paranasal sinuses of asymptomatic individuals, it is also important to realize that a significant portion of asymptomatic patients show abnormalities on CT scans [2].

When comparing concordance between endoscopy and CT staging in rhinosinusitis, the correlation was 65% for positive and 71% for negative results [118]. The various staging systems have been used to judge the severity of rhinosinusitis based on extent of the inflammatory disease within the paranasal sinuses. Most of the CT staging systems tried to divide severity into 4 grades or 4 stages (Kennedy, Levine and May, Friedman, Harvard). However, validation studies of the different staging systems have shown that a simple scoring system such as the Lund-Mackay score would better quantify severity of the disease, although no system currently available allows clinicians to judge the evolution of this disease or to indicate prognosis [119]. The Lund Mackay system is based on scoring each sinus with 0-2 points (0- no pathology, 1 point any partial opacity, 2 points- total opacity), giving a score of 0-12 per side. However, even this system does not result in significant correlation with symptom severity scores. Normal Lund-Mackay score for adults is 4.26 (95% CI, 3.43 to 5.10) [120] and for children it is 2,81 (95% confidence interval, 2.40 to 3.22), with only 19,3% having a score of 0 [119].

The accuracy of CT in the diagnosis of CRS was tested, comparing CT scores with histopathologic grade of inflammation and compared to a control group without rhinosinusitis utilizing well designed criteria. By using the ROC method, the sensitivity of CT was found to be 94% and specificity 41% using Lund score cut-off value for RS greater than 2, while putting it at the level of incidental Lund scores (above 4) increased specificity to 59%. Using the same method in pediatric rhinosinusitis, the same author found, using a Lund score cut off of five to represent true disease, sensitivity of the CT at 86% and specificity 85%. Lund scores of two or less have an excellent negative predictive value, and Lund scores of five or more have an excellent positive predictive value, strongly indicating true disease.

Plain sinus radiographs have shown poor sensitivity and specificity, so that even low irradiation does not justify its' use. In the studies comparing plain sinus radiographs with sinus CT scans, the sensitivity of plain film radiography ranged from 36.7 to 66.4% depending on localization, while specificity was high (90% and over) with the exception of the maxillary sinus (82%) in one study. The other study has confirmed better matching of the CT scans with plain sinus radiographs for maxillary sinusitis (78%), but only 52% for the ethmoids [121].

### Outcomes

CT scans remain the gold standard for diagnosis of rhinosinusitis and for pre-operative evaluation of the sino-nasal anatomy. CT scoring has been used to show improvement in different treatment studies in ARS and CRS but the correlation of the symptom improvement rate with CT score improvement rate was often not significant. Due to the radiation dose, CT score improvement cannot be used as an outcome measure for ethical reasons. As a good correlation between scores on CT and MRI has been shown, MRI might be a more appropriate imaging outcome. MRI is useful for diagnosis and follow-up of patients with benign (eg inverted papilloma) or malignant (eg adenocarcinoma) sinonasal pathology.

### Recommendations

There is no evidence to support the use of imaging in uncomplicated acute rhinosinusitis. In contrast to ARS where CT scans are not mandatory for diagnosis, CT scans may confirm the clinical diagnosis of CRS. The use of CT scans in 3-dimensional views are highly recommended as a roadmap for endoscopic sinus surgery in order to define specific anatomic relations in the individual patient.

Plain sinus radiographs have low informative value as is the case for ultrasonography, both being characterized by low sensitivity, but somewhat better specificity in maxillary and frontal sinusitis.

## Diagnosis of Occupational Rhinitis

### History

**Figure 17:**
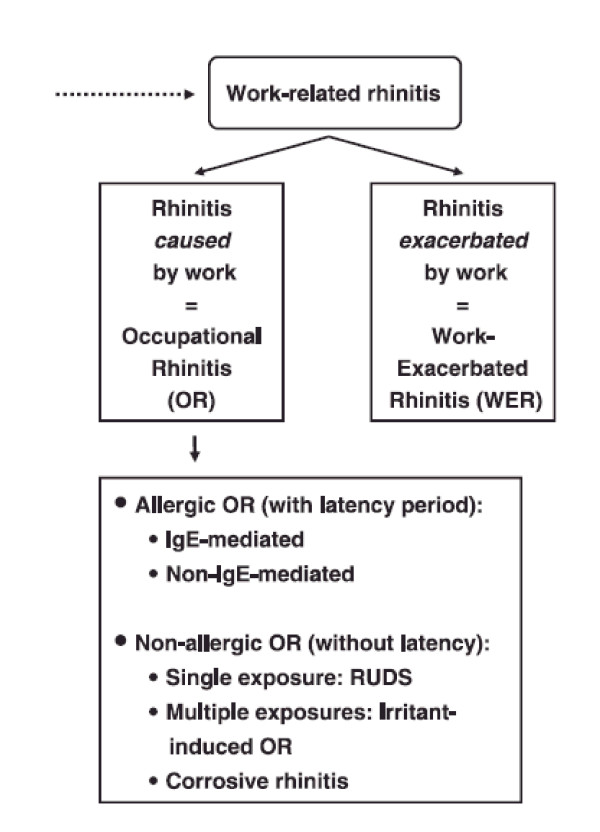
**Occupational vs work-exacerbated rhinitis**.

The diagnosis of occupational rhinitis (OR) is based on very careful and detailed medical history and history of exposure conditions at work [122]. The purpose of the clinical history is to confirm the existence of rhinitis and to evaluate it's the link to work. Because often the diagnosis of OR have substantial financial and work-related consequences, the relationship with the work exposure needs to be ascertained with provocation tests [122]. A distinction is made on rhinitis caused by agents in the working environment, i.e. OR, and rhinitis exacerbated by the work environment, i.e. work-exacerbated rhinitis (Figure 17).

### Symptoms and medical history

The medical history should include the information about the exposures at work and their potential capacity to cause sensitization or irritate the nasal mucosa. This information is normally asked from the employer. Any chemicals and proteins that can be sensitizers or irritants should be recorded. How they are used and what is the protective measure at work should be recorded. Patients should be asked about the time of the exposure starting, how long it has lasted and what is the exposure level and time of exposure daily and weekly. The relation of the symptoms to work and the alleviation of them when away from the work are important clues to diagnose OR. The typical symptoms are rhinorrhoea, sniffing, nasal stuffiness and nasal itch as in any allergic rhinitis but even one of these symptoms can be present, especially if the exposure has continue long. Often nasal stuffiness is the main symptom and rhinorrhoea and sneezing often subsides when the exposure continues longer timer. Patients can have hoarseness and coughing, even asthma symptoms during the work hours as well. It is relevant to ask when the patient first time was treated by physician because of rhinitis symptoms and also exclude other reasons for rhinitis like sinusitis and seasonal or perennial allergy.

### Examinations and immunological tests

A routine ENT examination including nasal endoscopy should be performed. Skin prick test and/or specific IgE tests for common aeroallergens and work related allergens and chemicals are mandatory. Common aeroallergens are tested to exclude their role and show if the patient is atopic already. It is know that atopic patients have more symptoms and are easier sensitized to new allergens. SPT are considered to be positive when the mean wheal diameter is exceeds 3 mm (area > 7 mm). Positive immunological test may appear in a substantial proportion of exposed asymptomatic individuals. On the other hand, a negative test result makes the diagnosis of OR unlikely, provided that appropriate allergens have been tested. The main limitation of immunological tests in the investigation of occupational allergy results from the lack of standardized, commercially available extracts, especially low molecular weight agents [122].

If lower airway symptoms like cough, wheezing, dyspnoea or diminished ability for physical strain exist, additional spirometry and other examinations to exclude asthma are needed. In addition, use of any medication and other airway or systemic diseases possibly relating to the symptoms should be recorded.

### Nasal and inhalation challenge tests

**Figure 18:**
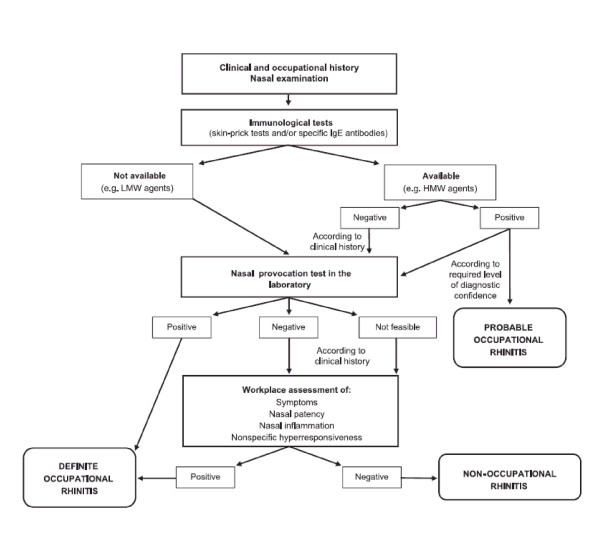
**Diagnostic algorithm for occupational rhinitis**.

Both nasal as well as bronchial challenge tests can be applied for the diagnosis of OR (Figure 18). Nasal challenge tests are the standard diagnostic tool to confirm the causative role of a specific agent in the development of rhinitis symptoms. Nasal provocation testing represents an essential tool in the diagnosis of allergic OR but needs to be evaluated in the context of the medical and work history and sensitization stat [123]. Several methods of exposure of one or both nostrils are being reported: throw dropping or spraying and solid agents by direct application, by special devices or by sniffing. Inhalation challenge (IC) tests are rarely reported to be used in the diagnosis of OR, but represent a valuable method in the simultaneous evaluation of patients with both lower and upper airways symptoms [124]. Even IC is resource-intensive methodology, the evaluation of nasal and bronchial reactions together save time and expenses compared to organization of multiple individual challenges [125].

There are no uniformly accepted criteria for the evaluation of nasal challenge reactions. In addition the test can be performed either unilaterally or bilaterally. Various symptoms and findings scoring criteria based on clinical findings or patient symptoms have been traditionally used as the main criteria of the positivity of nasal challenge reactions. For example the sneeze count, inspected nasal blockage (congestion), itching or burning of the nose, palate or throat and lacrimation have been used ^48 ^or symptom score of nasal itching, sneezing, and rhinoscopic nasal obstruction, rhinorrhea, and mucosal oedema [126]. In addition to the scoring criteria objective instruments like acoustic rhinometry and anterior rhinomanometry are used. Minimum cross-sectional area of the nose [127] and nasal volume from 2-5 cm depth have been used for evaluation of nasal mucosa reactions [128]. In addition nasal peak inspiratory flow as well as optical rhinometry and rhinostereometry have been introduced for the evaluation of nasal provocation results. Measurement of secretion has been introduced as one objective and relevant measurement of nasal provocation test, and that in a unilateral test it has shown to be slightly superior to acoustic rhinometry and rhinomanometry [129]. Sham provocation tests should be used to confirm the positive reaction with occupational exposure and exclude general nasal hyperreactivity.

### Recommendations

The diagnosis of occupational rhinitis is based on the patients' history. Allergic OR should be dealt with diagnostically like any other allergic rhinitis, whereas non-allergic occupational disease requires more specific attention. In case of important socio-economic impact of the diagnosis of OR, the diagnosis can be confirmed by provocation tests that need to be performed in a standardized way involving subjective as well as objective evaluation of inflammation.

## Future Tools

We are dealing with complex diseases in Rhinology and need a systems biological approach that could spell the end of clinical and basic science as independent research fields. Why do patients with the same sensitization present with such a variety of symptoms or why are some patients sensitized only for house dust mite or grass pollen, while yet others are sensitized for both? Are we looking at a single disease with different levels of expression or should we consider that these individuals suffer from similar, yet different diseases? There are a large number of chronic rhinosinusitis patients who may all suffer from the same disease or from different disease modalities that have a similar symptomatology. Clinical or basic scientific conclusions drawn from an inhomogeneous group of patients may misinterpret the molecular mechanisms or the clinical symptomatology of the disease under investigation.

From a clinical point of view it may seem that the healthy individual is probably the least interesting to study except as a control in all good studies, but hardly ever as the prime focus of research. This practice needs to be re-examined. It is very important to understand the normal healthy response and compare this response to the aberrant response in an allergic individual. In the case of the immunologic response this concept would lead to a number of states of the immune system that would also depend on whether we look at a healthy or a diseased individual. Moreover, these differences would not only be there at the tissue level, but also at the protein expression pattern level in individual cells.

We therefore need to re-evaluate our research tools. In hypothesis-driven research we investigate the role of one or a limited number of players on a complex disease process, limiting our powers of observation. New analysis tools may allow us to analyse a disease in a single individual. However this will mean that we need to collect many data points for a single patient, in contrasts to the present custom of collecting a few data points for many patients. Fortunately, any of the "omics" techniques (genomics, transcriptomics, or proteomics) allow this approach.

Genetics, transcriptomics, and proteomics are traditionally seen as three complementary and equivalent research fields in that each addresses a different part of the information highway. An important realization is that both the diseased and the healthy state is a complex interplay between a large number of factors that influence each other. The transition from healthy to diseased state could affect only a small proportion of the factors defining the healthy state, but current data suggest that differences are rather large. This is most clearly seen when the healthy and diseased states are compared at the tissue level. The question arises how the two states can change from one to the other. Due to the inherent presence of negative and positive feedback loops, a network is often very resistant to change. It may accommodate small changes, but large deviations from equilibrium may require more than one defect in network components. There are likely to be multiple different combinations of defects yielding a similar change from baseline. When a disease is studied by looking for defects on a genome wide level, we might run into the problem that although we study a single disease that the multitude of different combination of factors involved would introduce such a large mix that statistics may not be able to identify individual factors. At the moment it would seem that (A) a lot of factors have been reported to be affected and that (B) none of the factors are specific for the disease and are at best found enriched in the diseased population. Although impractical (one would need to include too many well-defined patients), the different studies into genetics should also consider previously collected data on other genes.

This particular problem does not arise when proteomics or transcriptomics is used, as the expression pattern will define the disease even though the underlying reasons for the change into the diseased state might be different. This last point is again one of the two biggest problems. The disease can be well-described using proteomics and transcriptomics, but now we will find it hard to define the mutation responsible for the diseased state. A concept that has not yet been put to the test would combine the best of both worlds; use transcriptomics and proteomics to define the disease and then focus the mutation analysis specifically on the genes/proteins that are found affected. This would reduce the number of genes to screen down to a more manageable level, so that perhaps even interactions between mutations in different genes would become feasible. A second down-side of transcriptomics and proteomics is in the selection of material that will be studied. Given the concept of systems biology it would be tempting to investigate diseased versus healthy tissue samples, but this comes at a price. Firstly, it is not always clear what needs to be compared. In the case of chronic rhinosinusitis one might question if healthy turbinate would be useful in the comparison with nasal polyps as, firstly, they are not identical structures and, secondly, they are found at different locations within the nasal cavity. Even when comparable tissues can be found (eg. turbinates of allergic and healthy individuals) transcriptomics and proteomics would also measure differences in tissue composition. Techniques that would take this into account are struggling to discriminate between changes in expression in a given cell and difference caused by differences in numbers of these cells between diseased and healthy tissues. Focusing on a single cell type would eliminate this problem, at the cost of a less comprehensive overview of the systems biology and the unknown consequences on expression patterns in cells that have been isolated and as a consequence have been taken out of their normal tissue environment.

### Dealing with complex data

After all the clinical and molecular data have been collected, there is a long list of matters that differ between a healthy and a diseased individual. We could analyze the items on the top of the list that have changed the most in more detail, but it would seem to defeat the point of collecting all data in the first place. We do not know whether items that change the most, are also the most important in the disease process. A number of analytical tools exists that can help our understanding [130]. The first tool helps us to define what causes the variation in all the data we have collected. Principal component analysis lists these causes in order of their contribution. An example from our own epithelial work in allergic rhinitis shows that the diseased state (the difference between healthy and allergic) is the most important component, followed by differences between healthy individuals [131]. This reminds us again that we are all clearly different, but also shows that the allergic individuals resemble each other more strongly. Interestingly, this approach could also be used as a diagnostic tool as the location in the plot could identify the individual as either diseased or healthy. Moreover should we have labelled an individual incorrectly, this would not have affected the outcome as individuals are separate data points in the graph and need not to be grouped beforehand for this analysis.

The second tool is network analysis showing how the factors we have identified interact together. Using data from literature it either groups the items on the basis of previously reported physical interaction or on reported effects of one item on the expression pattern of another. Again from our own work on the effect of house dust mite allergen on nasal epithelial cells we derive a transcription regulation model [131,132]. This can be combined with a clustering tool that describes the expression behaviour of genes. At this last level we can think of interfering with the disease as the type of expression profile in a cluster might represent an essential behaviour to deal with the diseased state. Understanding the regulation of such a cluster of the transcription factor level, the signal transduction level, or the recently identified micro RNA level, could lead to the identification of new targets for treatment.

## Concluding Recommendations

In the following table (table 7), recommendations for each diagnostic tool which has been revised are summarized.

**Table 7:** Recommendations for diagnostic tools in rhinoloy

	AIMS	METHODS INSTRUMENTS	RECOMMENDATIONS
HISTORY	Evaluation of * patients' symptoms and symptom severity * co-morbidities and general medical condition * medical/surgical history * exposure to allergens/irritants * cigarette smoke	* Personal communication * Questionnaires	Essential part of diagnostic process In all patients with nasal problems.... and in those with lower respiratory tract disease!

QoL TOOLS	Evaluation of the impact of nasal disease on * quality of life * different domains of physical and mental functioning	* Generic * Disease-specific	Helpful in clinical practice and clinical trials

NASAL EXAMINATION	Evaluation of the * external and endonasal anatomy * endonasal mucosa and lumen	* Inspection * Palpation * Ant. and post. rhinoscopy * Nasal endoscopy	* Non-ENT doctors should examine the nose including ant. rhinoscopy * A nasal endoscopy is recommended in chronic rhinologic disease

ALLERGY TEST	Evaluation of the sensitization state, including the demonstration of the specific sensitization state	* Skin prick test * Blood analysis with allergen-specific IgE	Recommended in all patients with clinical suspicion of allergic AW disease

NASAL PROVOCATION TEST	Evaluation of the response of the nasal mucosa to * allergens * aspirin * occupational agents	Provocation by inhalation, spray, nasal drop or discette	Recommended in case of doubt about sensitization

SMELL TEST	Evaluation of the smell capacity	Different tests are currently available	Recommended in case of severe hyposmia or anosmia

TASTE TEST	Evaluation of taste capacity	Electrogustometry	Recommended in patients with taste dysfunction

NASAL PATENCY MEASUREMENTS	Evaluation of a patients' capacity to breathe through the nose	* PNIF * Anterior rhinomanometry * Acoustic rhinometry	Recommended parameter in clinical trials Helpful in clinical practise to evaluate the evolution of nasal patency

NO measurement	Evaluation of NO levels in nasal cavity	Chemiluminiscence reaction of expired air	Helpful as screening tool in PCD

NASAL SAMPLING	Collection of nasal mucosa/cells/secretions for analysis	* Nasal secretions * Nasal scraping * Nasal biopsy	Recommendations: * nasal sampling in experimental/clinical studies * nasal secretions for B2 transferrin analysis in suspicion of CSF leak * biopsy in case of unilateral/malignant disease

BLOOD AND ADDITIONAL TESTS	Evaluation of the sensitization state, immune system, endocrine system Evaluation of mucociliary function Evalution of chloride content in sweat	* Blood test * MCT, nasal NO, EM, ciliogenesis in vitro * Sweat test	Recommended as diagnostic tool in severe rhinitis, rhinosinusitis and nasal polyp disease with suspicion of underlying auto-immune, immunologic or ciliary disease

## Summary

We need to merge all available data into one big model of reality. The second important issue is that we need to be very strict in our patient selection as failure to do so will yield confusing data or alternatively focus on a single individual as if he or she has unique disease, and then try to cure this disease for this individual. The unification of clinical and basic scientific research has started and will be with us for some time to come.

## Appendix

### Different Tools to Assess Sense of Smell

#### University of Pennsylvania Smell Identification Test (UPSIT)

Method: The UPSIT test is a rapid and easy-to-administer method to quantitatively assess human olfactory function [133]. The UPSIT shows a high test-retest reliability and scores on this test are strongly correlated with the detection threshold for phenyl ethyl alcohol in the same individuals. When the UPSIT is administered in the standardized manner, clinical subjects show a high degree of uniformity in UPSIT performance when tested in different laboratories. The 40-odorant UPSIT is used in over 1500 clinics and laboratories throughout the United States, Canada, South America, and Europe, and has been administered to nearly 200,000 people since its development in the early 1980s. Hundreds of published papers have employed this test in academic, clinical, and industrial settings. A particular strength of this test is that it provides an olfactory diagnosis based on comparing the patient's test score with normative data, providing a percentile score of an individual relative to his or her age-matched normal group. Furthermore, a clinician can distinguish patients with a normal sense of smell ("normosmia") from those with different levels of reduction ("mild, moderate and severe microsmia") or loss ("anosmia"). The test can also distinguish "probable malingerers" from those with true olfactory deficits.

The test consists of four booklets, each containing 10 odorants with one odorant per page. The stimuli are embedded in 10-50 (mu) diameter plastic microcapsules on brown strips at the bottom of each page. Above each odorant strip is a multiple-choice question with four alternative words to describe the odour. The subject was asked to release the odorant by rubbing the brown-strip with the tip of a pencil and to indicate which of four words best describes the odour. Thus each subject received a score out of 40 possible correct answers.

Test-time: 15 min.

Test-retest reliability: r: 0.981.

Country: USA

#### Conneticut Chemosensory Clinical Research Center Test (CCCRC)

Method: The CCRC consists of two tests of olfactory performance [134]: an odour identification task and the determination of the n-butanol threshold. Presentation of odorants for threshold testing is performed by means of a plastic squeeze bottles while odour identification is assessed by means of sniff bottles (bottles made of glass). The highest concentration of butanol in the series was 4% in water; 11 successive dilutions were established as a geometric series dilution ratio of 1:3. Testing was performed with the concentrations in ascending series using a two-alternative, forced choice paradigm by which patients have to identify the odorant containing bottle after both odorant and blank have been administered (double-alternative, forced choice paradigm). The threshold was defined as the concentration at which subjects correctly identify n-butanol in five successive trials. The odour identification task employed eight items (baby powder, chocolate, cinnamon, coffee, mothballs, peanut butter, ivory soap and Vicks Vaposteam). Patients were given a list with 16 terms comprising eight terms describing the items used in the test and eight items describing other common items. All odorants were handled most carefully; the experimenters always wore deodorized disposable cotton gloves. Measurements were performed in quiet, well-ventilated rooms.

Test-time: 35 min.

Test-retest reliability: not found.

Country: USA

#### Smell diskettes test

Method: A screening test of olfaction was designed [135] using 8 diskettes containing different odorants (coffee, vanilla, smoke, peach, pineapple, rose, coconut, vinegar). These diskettes (5 × 6 cm) are widely used in the perfume and flavour industry as applicators for odorants. The odorants were used in a high suprathreshold concentration. The test was designed as a triple forced multiple choice test, resulting in a score from 0 to 8 correct answers. The answers were presented on a questionnaire with illustrations.

Test-time: 5 min.

Test-retest reliability: r = 0.99

Country: Zürich, Switzerland

#### Odourant confusion matrix

Method: Odorant identification test in which the number of correct odorant identifications quantifies the level of olfactory function [136]. The OCM reflects distortions of sensory perception as errors in identification. Subjects attempted to identify each of 10 odorant (ammonia, cinnamon, licorice, mint, mothballs, orange, rose, rubbing alcohol, vanilla, vinegar, vex) stimuli (plus a blank known as a vex) from a list of 10 odorant names, which was visible at all times. Subjects were not told of the presence of the vex, and were asked to respond to all stimuli with an odor name chosen from the list of names. The 11 stimuli were given to the subject in 11 randomized blocks, resulting in a total of 121 stimulus presentations [136].

Test-time: 15 min.

Test-retest reliability: not found.

Country: USA

#### Dutch odour identification test (GITU)

Method: Identification of 18 or 36 odours in jars. Forced choice either from 4 alternatives or from a list of 24 for 18 odours to identify [137].

Test-time: not found.

Test-retest reliability: not found.

Subject differences:

Country: The Netherlands

#### YN-odour Identification Test (YN-OIT)

Methods: identification was assessed with a four alternative-forced-choice task modified from the UPSIT odours and a yes/no task yielding measures of discrimination and response bias for the same stimulus material [138].

Test-time: not found

Test-retest: not found

Country: USA

#### T&T Olfactometer

Methods: The T&T olfactometer is the most commonly method used in patients suffering from smelling disorders. The T&T olfactometer (Daiichi-Yakuhin, Tokyo, Japan) consists of five test odorants [139]. Each odorant was diluted into eight log-step concentration series with either propylene glycol or liquid paraffin (grade 5 to -2). The detection threshold is the weakest concentration at which the stimulus is firstly noticed. The concentration at which a qualitative sensation is first recognized is recorded as the recognition threshold.

Test-time: not found

Test-retest: not found

Country: Japan

#### San Diego Odor Identification Test (SDOIT)

Methods: Eight-item odour blind identification test [140] that uses common odours typically found in the home (chocolate, coffee, etc). Odorants were wrapped in gauze and kept in opaque containers to minimize visual clues. The inter-stimulus interval was 45 seconds to minimize adaptation. A picture board with illustrations of the target items (8) as well as distracters (12) was presented to aid in identification. The odorants were presented in random order to the participant

Test-time: not found

Test-retest: r: 0.86

Country: USA

#### Cross-Cultural Smell Identification Test (CC-SIT)

Methods: 12-item self-administered micro-encapsuled odour identification test, analogous to the UPSIT that incorporates multicultural odorant items selected from the UPSIT that are familiar to most persons from North America, European, South American, and Asian cultures [141].

Test-time: <5 min.

Test-retest: r: 0.71

Country: USA, Europe, Asia

#### Combined olfactory test (COT)

Robson et al. [142] creates the COT developed a screening test to evaluate the olfaction in adults and children.

Method: Threshold with n-butanol in plastic containers, and identification of 9 odours in jars with forced choice with 4 options.

Test-time: not found

Test-retest reliability: not found

Country: UK, New Zealand

#### Sniffin'-Sticks

Method: Test of nasal chemosensory performance based on pen-like odour dispensing devices [143]. For evaluation of odour sensation, the cap was removed by the clinician for 3 seconds and the pen's tip placed 2 cm from both nostrils. It comprises three tests of olfactory function, namely tests for odour threshold (n-butanol by means of a single staircase), odour discrimination (16 pairs of odorants, triple forced choice), and odour identification (16 common odorants, multiple forced choice from four verbal items per test odorant). Criteria for the selection of odorants were as follows: 1. subjects should be familiar with all odor-describing items used in the test; 2. odorants included in the test should be similar with regard to both intensity and hedonic tone; and 3. the successful identification of individual odorants from a list of four descriptors should be >75% in healthy subjects.

Test-time: 25 min.

Test-retest reliability: r: 0.61, r: 0.54, and r: 0.73 for threshold, discrimination, and identification respectively.

Country: Germany, USA

#### Candy smell test (CST)

Method: an easy-to-use, reliable, and fast test of retronasal olfactory performance (23 aromatized sorbitol candies using a four-alternative with forced-choice procedure) suitable for the screening of smell function in children above the age of 6 years and adults [144].

Test-time: 5 minutes

Test-retest reliability: r = 0.75.

Country: Germany.

#### Alcohol Sniff Test (AST)

Method: In 1997, Davidson and Murphy developed a screening test to evaluate the olfaction in adults and children [145]. The AST is a rapidly administered and employs odour material readily available in the medical environment, providing a measure of first cranial nerve activity. A standard 70% isopropyl alcohol prep pad was opened such that 0.5 cm of the pad itself was visible. The alcohol pad was placed beneath the nostrils while the subject inspired to familiarize with the alcohol odour. Active sniffing and deep inspiration were discouraged. Then the alcohol pad was placer 30 cm below the nose and with each inspiration the pad is placed 1com closer to the nose until the subject detected the presence of the odour. The distance from the anterior nostrils to the alcohol was measured in cm. The procedure was repeated five times and the mean distance defined the threshold.

Test-time: 5 minutes

Test-retest reliability: r = 0.8.

Country: USA

#### Culturally Adjusted University of Pennsylvania Smell Identification Test (CA-UPSIT)

Method: Ahlskog et al. developed a culturally adjusted olfactory test battery, derived from the original UPSIT for clinical use on the Chamorro inhabitants of Guam, a western Pacific island [146]. Identification of 20 microencapsulated odours with the "scratch and sniff" technique. Each of the 20 odours has four response alternatives. Target odours included: smoke, lilac, lemon, bubble gum, motor oil, banana, leather, coconut, onion, gasoline, peanut, dill pickle, lime, watermelon, grass, soap, cherry, strawberry, root beer, and mint.

Test-time: not found

Test-retest reliability: not found.

Country: USA

#### Kremer et al [147]

Method: Olfactory test using spray bottles. The atomizer bottles were made of glass and contained 12.5 ml (10 g) of fluid. A special administration valve limited the amount of solution set free with each application to exactly 2 mg. The even distribution of the expelled solution was assured by the extremely fine atomization produced. The average size of the aerosol was 40 μm. For hygienic reasons a replaceable valve was used. To implement the olfactory test using spray bottles the patient was required to hold the atomizer 5 cm before an opened mouth. The spray was administered during inspiration, and afterwards the patient was asked to exhale through the nose, keeping the mouth closed. Alternatively, the solution colud be sprayed into the lid of the bottleand then be sniffed. Between separate applications the patient was given a rest period of at least 30 seconds. The substances in the olfactory spray test were: rose, cinnamon, banana, pine essence, tangerine, and peppermint. The spray test was shown to be easily performed and was suitable as a screening test, with a sensitivity of 88% and a specificity of 100%.

Test-time: 4 minutes

Test-retest reliability: not found

Country: Germany, The Netherlands

#### Scandinavian Odour-Identification Test (SOIT)

Method: Nordin et al. [148] developed this olfactory test to address the need for a culturally valid odour-identification test for clinical use on the Scandinavian population that has good ability to generalize performance to olfactory status, assess olfactory and trigeminal function separately with a good sensitivity and specificity. 16 odours (`pine needle, peppermint, juniper berry, violet, orange, cinnamon, lilac, apple, lemon, tar, anise, vanilla, bitter almond, clove, ammonia) to assess both cranial nerves. The stimulus order was randomize for each participant (>600). The liquid odorant was injected into a tampon filled to saturation and placed in an opaque, 80 ml glass jar. For each stimulus the participant was provided with a written list of four response alternatives from which to choose the most appropriate item for identification. The stimuli were presented birhinally, 5 cm under the nose, for as long as required to accomplish the task.

Test-time: 15 minutes

Test-retest reliability: r = 0.79.

Country: Sweden, Finland

#### Pocket Smell Test [56]

Method: A screening measure of odour identification derived from the University of Pennsylvania Smell Identification Test (UPSIT) with identification of 3 microencapsulated odours with the "scratch and sniff" technique. Each of the three PST tasks contains four response alternatives [149]. Target odours included: smoke, lilac, and lemon. After releasing the odour, the examiner read the four response alternatives continuously until the patient responded. Patients were encouraged to guess if they were unsure. Nostrils were not tested separately.

Sample size: 140

Country: USA

#### Eloit and Trotier Olfactory Test

Methods: computed-assisted olfactory test to measure thresholds, detection, and identification using 5 odours: phenyl-ethyl-alcohol (PEA; flowers, rose, jasmine), cyclotene (CYC; caramel, cake), isovaleric acid (IVA; fruits, apricot, peach), undecalactone (UND; cowshed, slurry), and skatole (SKA; faecal). These chemically stable substances in bottles evoke different odour notes. A software was developed to assist in the procedure of the experiment. At the beginning, participants get accustomed with the 5 odours at the highest concentration and learned to name them. Every odour was divided in 7 different descending concentrations. For each bottle, the subject was asked to decide whether it contained an odorant and, if so, which odorant was present. For each odorant, the detection level was taken as the highest concentration not perceived plus one. The identification level was taken as the smallest concentration correctly identified in a series of correct identification beginning with the highest concentration [150].

Test-time: not found

Test-retest: not found

Country: France

#### Ramdon Test

Method: a labelling of 16 concentrations of two odorants (citrus, rose) randomly presented for thresholds, discrimination, and identification [151]. Subjects were blindfolded to prevent visual identification of the odour-containing pens.

Test-time: 10 minutes

Test-retest reliability: r = 0.71.

Country: Germany

#### Four-minute odour identification test

Method: Screening test on the basis of the odour identification test as used in the "Sniffin' Sticks" olfactory test [152]. Identification of 12 microencapsulated odours in pens. For odour presentation the cap is removed by the experimenter for approximately 3 seconds and the pen's tip is placed approximately 2 cm in front of both nostrils. Identification of individual odorants was performed from a list of 4 descriptors each. Target odours included: cinnamon, banana, lemon, licorice, pineapple, coffe, cloves, rose, leather, fish, orange, and peppermint. Nostrils can be tested separately.

Test-time: 4 minutes

Test-retest reliability: r = 0.78.

Country: Germany

#### Barcelona Smell Test (BAST-24)

Method: Olfactory test addressed to the need for a culturally valid odour-identification test for clinical use on the Spanish population and in general for the Mediterranean countries. 24 odours, 20 odours (banana, gasoline, lemon, rose, onion, smoked, anise, coconut, vanilla, melon, mandarin, bitter almond, pineapple, cheese, strawberry, mushroom, eucalyptol, clove, turpentine, and peach) to assess the 1st cranial nerve and 4 odours (vinegar, formol, mustard, ammonia) to assess the 5th cranial nerve. Hermetic containers were designed to contain the different odorants in a semi-solid state. BAST-24 scores smell detection, identification, and forced choice. Nostrils can be tested separately, smell identification was scored slightly higher in the left than in the right nostril for both cranial nerve. BAST-24 was found to be a valid, reliable, and reproducible test [153].

Test-time: 20 minutes.

Test-retest reliability:

Country: Spain

#### Nez du Vin smell test

Six odours initially derived from a kit used to educate wine tasters presented on paper strips with a forced choice between 4 possibilities.

Time: 2 minutes

Test retest -correlation with UPSIT: r = 0.79, p < 0.001

Country:UK

#### Nez du Vin smell test

Six odours initially derived from a kit used to educate wine tasters presented on paper strips with a forced choice between 4 possibilities [154]. Test-time: 2 min. Test retest -correlation with UPSIT: r = 0.79, p < 0.001. Country:UK

## Consent

Consent for publication of photos was obtained from all pictured patients or their relatives.

## Abbreviations

AR: Allergic rhinitis; ASNC: Allergen-specific nasal challenge test; CF: Cystic fibrosis; CRS: Chronic rhinosinusitis; CT scan: Computerized Tomography scan; MRI: Magnetic resonance imaging; NL: Nasal lavage; NO: Nitric oxicde; NP: Nasal polyps; PCD: Primay ciliary dyskinesia; PNIF: Peak nasal inspiratory flow.

## Competing interests

All authors declare they have no competing interests.

## Authors' contributions

History of patient by GS; nasal clinical examination by PH; allergy tests and nasal provocation tests by GP; assessing the senses of smell and taste by JM, JG and IA; microbiology by LK; blood tests by GS; imaging in rhinology by LK and VL; evaluation of nasal patency, nasal peak flow, rhinomanometry, acoustic rhinometry by PH; quality of life assessment in rhinosinusitis, patient reported outcomes, rhinitis control tests by RGvW; nasal air sampling, NO measurement by GS; nasal cellular sampling, lavages, cytology, biopsies by PG and CB; evaluation of mucociliary transport by PH; diagnosis of occupational rhinitis by ET; future needs in clinical and research tools in rhinology by WF and CvD; concluding remarks and recommendations by GS and PH. All authors read and approved the final manuscript.
